# ﻿The ants of the Galápagos Islands (Hymenoptera, Formicidae): a historical overview, checklist, and identification key

**DOI:** 10.3897/zookeys.1191.107324

**Published:** 2024-02-13

**Authors:** Henri W. Herrera, María C. Tocora, Gianpiero Fiorentino, Charlotte E. Causton, Wouter Dekoninck, Frederik Hendrickx

**Affiliations:** 1 Royal Belgian Institute of Natural Sciences, Vautierstraat 29, B-1000 Brussels, Belgium Royal Belgian Institute of Natural Sciences Brussels Belgium; 2 Charles Darwin Research Station, Charles Darwin Foundation, Puerto Ayora, Santa Cruz, Galápagos Islands, Ecuador Charles Darwin Research Station, Charles Darwin Foundation Puerto Ayora Ecuador; 3 Ghent University, Terrestrial Ecology Unit, K.L. Ledeganckstraat 25, 9000 Gent, Belgium Entomology Museum Riobamba Ecuador; 4 Entomology Museum, Escuela Superior Politécnica de Chimborazo, Facultad de Recursos Naturales, Panamericana Sur km 1½, Riobamba, Ecuador Ghent University Gent Belgium; 5 Department of Ecology and Evolutionary Biology, University of Toronto, Toronto, ON M5S 3B2, Canada University of Toronto Toronto Canada; 6 Department of Biological Sciences, Texas Tech University, Lubbock, TX, USA Texas Tech University Lubbock United States of America; 7 Department of Biological Sciences, New Jersey Institute of Technology, Dr. Martin Luther King Jr Boulevard, Newark, NJ 07102, USA New Jersey Institute of Technology Newark United States of America

**Keywords:** Checklist, distribution, Galápagos ants, taxonomy

## Abstract

The Galápagos ant fauna has long been understudied, with the last taxonomic summary being published almost a century ago. Here, a comprehensive and updated overview of the known ant species of the Galápagos Islands is provided with updated species distributions. The list is based on an extensive review of literature, the identification of more than 382,000 specimens deposited in different entomological collections, and recent expeditions to the islands. The ant fauna is composed of five subfamilies (Dolichoderinae, Dorylinae, Formicinae, Myrmicinae, and Ponerinae), 22 genera, 50 species, and 25 subspecies, although three species (*Crematogastercrinosa* Mayr, 1862, *Camponotussenex* (Smith, 1858), and *Solenopsissaevissima* (Smith, 1855)) are considered dubious records. Finally, an illustrated identification key of the species found in the archipelago is presented.

## ﻿Introduction

**Figure 1.**
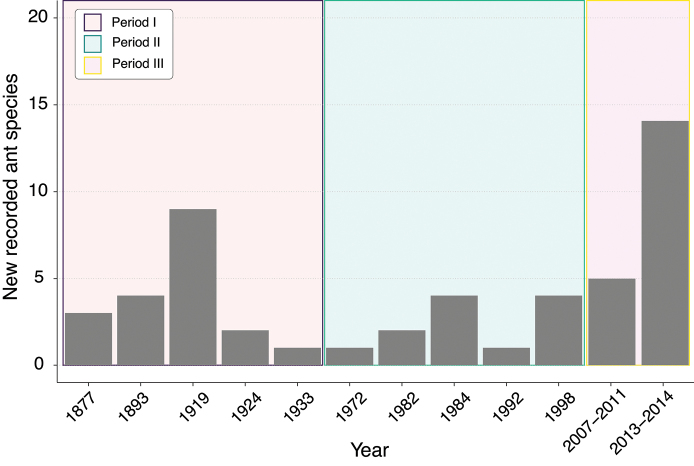
Delimitation of the three periods in the study of Galápagos ants. Period I: 1877–1933; Period II: 1933 until the end of the 1990’s; Period III: 2000 onwards. The following references correspond to the years listed in the figure: 1877 = Smith (1877); 1893 = Emery (1893); 1919 = Wheeler (1919); 1924 = Wheeler (1924); 1933 = Wheeler (1933); 1972 = Silberglied (1972); 1982 = Clark et al. (1982); 1984 = Lubin (1984); 1992 = (Snelling and Longino 1992); 1998 = Pezzatti et al. (1998); 2007 – 2011 = Pacheco et al. (2007), Herrera and Longino (2008), Herrera and Causton (2010), Lattke (2011); 2013 – 2014 = Herrera et al. (2013, 2014).

Until recently, the ant fauna of the Galápagos Islands was poorly studied. Early expeditions to the Galápagos collected only a few specimens at specific sites, primarily in the arid zones, which were more accessible (Smith 1877; Emery 1893; Wheeler 1919, 1924, 1933). This resulted in the first lists of Galápagos ant species published by Wheeler (1919, 1924, 1933) and Stitz (1932). Linsley and Usinger (1966) updated these lists by compiling all known reports of ants in the Galápagos archipelago, reporting 19 species and 34 subspecies. Only after Silberglied (1972) reported *Wasmanniaauropunctata* (Roger, 1863), the invasive little fire ant, in the Galápagos, did the interest in ants increase. At that time, studies were mainly focused on understanding the impact of *W.auropunctata* on native species (Clark et al. 1982; Lubin 1984). These studies led to several new records though they were limited to certain localities on a few major islands. Later studies by Pezzatti et al. (1998) and Snelling and Longino (1992) provided some important additions to the Galápagos ant fauna, but a systematic sampling of all islands was still needed (Brandão and Paiva 1994). In 2005, we initiated a project to study material deposited in collections worldwide and sampled all major islands in the archipelago, which resulted in many new ant records (Fig. 1) (among others: Longino 2003; Pacheco et al. 2007; Herrera and Longino 2008; Herrera and Causton 2010; Lattke 2011; Herrera et al. 2013, 2014). Here, we list all known species records (past and present) from the Galápagos Islands and provide an illustrated identification key for the established 47 taxa known to date, we do not include dubious records in the key. Also, this checklist does not include species intercepted in quarantine inspection activities in the Galápagos as these have not been confirmed as established in the islands. These intercepted species include: *Acromyrmexoctospinosus* (Reich, 1793), *Brachymyrmexpatagonicus* Mayr, 1868, *Camponotusbrettesi* Forel, 1899, *Crematogastercurvispinosa* Mayr, 1862, *Ecitonvagansangustatum* Roger, 1863, *Ectatommaruidum* (Roger, 1860), *Linepithemahumile* (Mayr, 1868), and *Notoncusectatommoides* (Forel, 1892).

## ﻿Materials and methods

**Figure 2.**
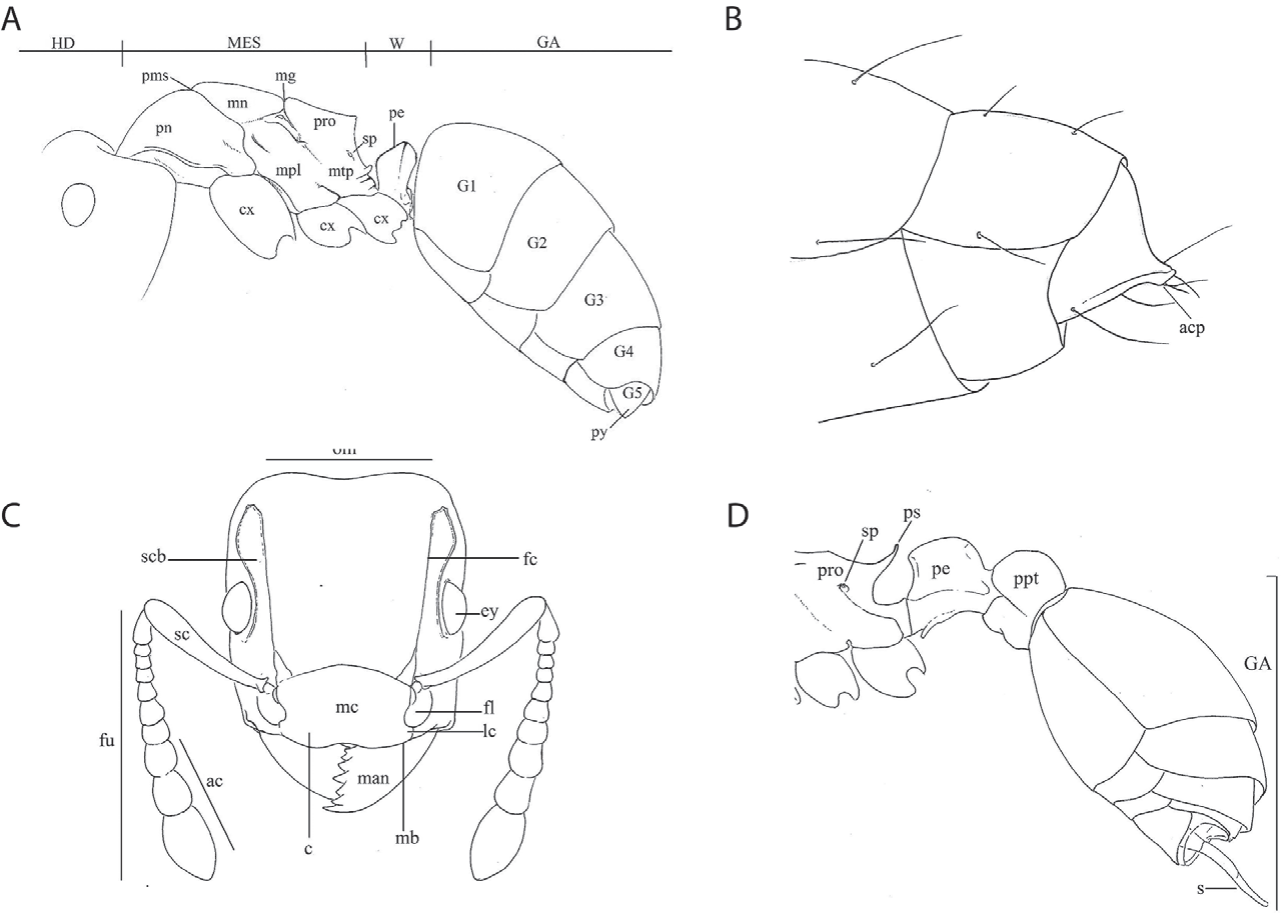
Glossary of terminology labeled from left to right **A** lateral view of a major worker of *Camponotusplanus***B** profile view of terminal portion of gaster of *Paratrechinalongicornis* (Latreille, 1802) **C** frontal view of *Tetramoriumbicarinatum* (Nylander, 1846) **D** lateral view of *Tetramoriumbicarinatum*. Abbreviations: ac = antenna club; acp = acidopore; c = clypeus; cx = coxa; ey = eye; fc = frontal carina; fl = frontal lobe; fu = funiculus; GA = gaster; G1, 2, 3, 4, 5 = gastral segments 1–5; HD = head; lc = lateral portion of clypeus; man = mandible; mb = basal margin of mandible; mc = median portion of clypeus; MES = mesosoma; mg = metanotal groove; mn = mesonotum; mpl = mesopleuron; mtp = metapleuron; om = occipital margin; pe = petiole; pms = promesonotal; pn = pronotum; pro = propodeum; ppt = post-petiole; ps = propodeal spine; py = pygidium; s = sting; sc = scape scb = scrobe; sp = spiracle.

This paper is based on literature reviews and the study of 382,023 specimens deposited mostly in the Terrestrial Invertebrates Collection of the Charles Darwin Research Station (**ICCDRS**) as well as the collections of John T. Longino (**JTLC**), California Academy of Sciences (**CAS**), Quito Catholic Zoology Museum (**QCAZ**), the University of Texas Insect Collection (**UTIC**) and the Royal Belgian Institute of Natural Sciences (**RBINS**). We mapped the geographical distribution of the sampling events using the Free and Open Source QGIS. We revised and updated information on samples used for previous publications and indicated where this material is deposited. The list of subfamilies and species is ordered alphabetically. Specimens from the genus *Nylanderia* are currently under revision and are merged into *Nylanderia* spp. Accordingly, only previous literature records of *Nylanderia* species are included in the checklist. We implemented a similar approach for the only known from Galápagos subspecies of *Camponotusmacilentus* Smith, 1877 and *Camponotusplanus* Smith, 1877, for which the taxonomic key is only at species level. Neither the material examined, nor the vague descriptions found in old literature allowed us to morphologically discriminate between the proposed subspecies. The genus *Nylanderia* in Galápagos and the *Camponotus* (sub)species complexes will be addressed in future studies. Scanning images at high resolution were obtained using Scanning Electron Microscope (SEM) (Todokoro and Ezumi 1999) and z-stacked images available in AntWeb (Herrera 2019) were used to illustrate the key. Morphological terms referred to in the key (Fig. 2A–D), followed Eady (1968), Harris (1979), Bolton (1994), and Bolton et al. (2003). Locality terminology referring to the different volcanoes on Isabela Island is as follows: Alcedo Crater (**CA**), Volcano Alcedo (**VA**), Volcano Ecuador (**VE**), Volcano Darwin (**VD**), Volcano Sierra Negra (**SN**), and Volcano Wolf (**VW**).

## ﻿Results

Five subfamilies of Formicidae can be found in the Galápagos: Dolichoderinae, Dorylinae, Formicinae, Myrmicinae, and Ponerinae, representing 22 genera, 50 species and 25 subspecies. The subfamily Myrmicinae is the largest with 32 species, while only one species on the islands represents Dorylinae, *Cylindromyrmexwhymperi* (Cameron, 1891). The introduced species *Solenopsisglobularia* (Smith, 1858) (on 35 islands, islets, and/or rocks), *Tetramoriumbicarinatum* (Nylander, 1846) (on 33), *Cardiocondylaemeryi* Forel, 1881 (on 30), *Monomoriumfloricola* (Jerdon, 1851) (on 27), *Camponotuszonatus* Emery, 1894 (on 24), *Tetramoriumlanuginosum* Mayr, 1870 (on 24), *Wasmanniaauropunctata* (on 21), *Solenopsisgeminata* (Fabricius, 1804) (on 20), and *Tapinomamelanocephalum* (Fabricius, 1793) (on 18), are the most widely distributed species in the archipelago. Among the putative endemic species (8, Herrera et al. 2020), *Leptogenyssantacruzi* Lattke, 2011 is most rare, with only a few records from the islands of Santa Cruz and Santiago.

## ﻿Discussion

We report 50 species and 25 subspecies of ants from 22 genera from the Galápagos Islands. The number of new species and locality records in the last 15 years combined with the fact that many islands are still highly understudied demonstrates that considerable work still needs to be done to identify and understand the islands’ ant diversity.

Of the species recorded in this checklist, there are still dubious records. This is the case for *Camponotussenex*, *Crematogastercrinosa*, and *Solenopsissaevissima* (Wheeler 1924; Crocker 1933; Peck et al. 1998). Recent fieldwork, extensive studies and revision of old collections could not confirm their presence in the archipelago. Wheeler (1924) defined *C.senex* as a species that is unlikely to be present in the Galápagos, while Trager (1991) and Pacheco et al. (2007) did not mention *S.saevissima* as part of the fauna of the archipelago. Regarding *C.crinosa*, this species could have been sampled from locations outside the archipelago by Mr. Maurice Willows during the Templeton Crocker Expedition (Crocker 1933). Wheeler (1924) cataloged this record as unexpected in the Galápagos. These three species are not included in the taxonomic keys in this work. Furthermore, the records of *Anoplolepisgracilipes* (Smith, 1857), *Camponotusplanatus* Roger, 1863, *Strumigenysgodeffroyi* Mayr, 1866, *Tetramoriumpacificum* Mayr, 1870, and *Pseudoponerastigma* (Fabricius, 1804) in the Galápagos (McGlynn 1999) are considered doubtful due to potential misidentification of these species. It is also possible that these species were collected on recently arriving in Galápagos and that they did not establish. As such, these species are not included in this species checklist.

Regarding the genus *Camponotus*, our studies suggest that the identification of the introduced ant *Camponotuszonatus* may have been confused with that of the only known from Galápagos species *C.macilentus*. This confusion is of particular interest regarding ecological studies that have cited the abundance of *C.macilentus*, which is typically more cryptic (McMullen 2011). Material examined retrospectively by the first author, collected by Pezzatti et al. (1998), von Aesch and Cherix (2005), and von Aesch (2006), showed that *C.zonatus* was collected during these field trips, nevertheless, this ant is not mentioned in any of these papers. Some of the records for the subspecies of *Camponotus* are also questioned for putative subspecies of *C.macilentus* and *C.planus* in the archipelago, and for now, we have only cited the records of Wheeler (1919, 1924, 1933) and Stitz (1932). Lastly, for the genus *Nylanderia*, future taxonomic and genetic studies are necessary to understand the number of species present and their status in Galápagos.

**Figure 3.**
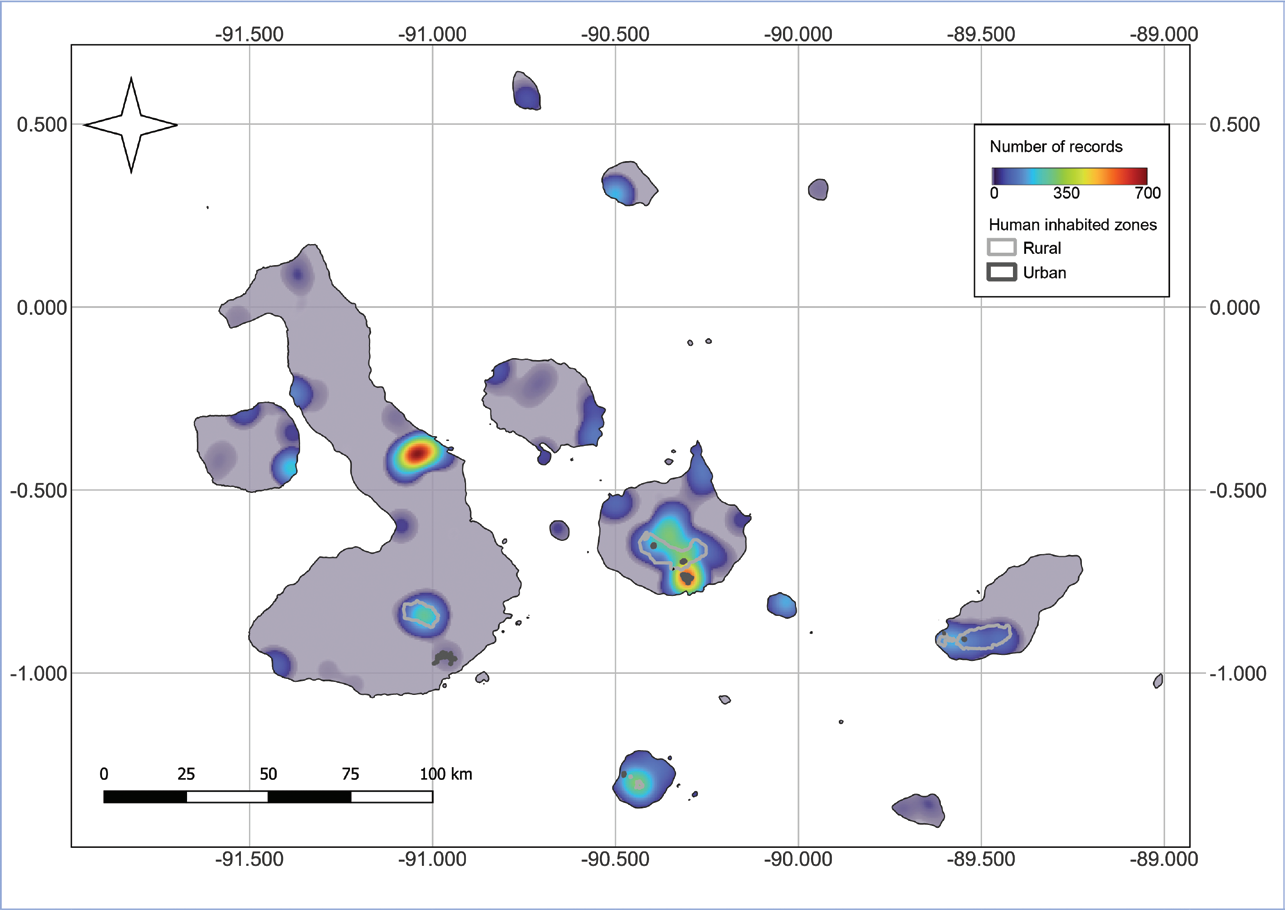
Heatmap highlighting the distribution of ant samples taken from 1963 to 2022. Regions indicated in pale purple have not yet been sampled for ants.

Although efforts in the last two decades have substantially increased our knowledge of the ant fauna of the Galápagos Islands, a good portion of the material studied during the last 15 years came from surveys that were not focused on ants. Ants remain poorly studied, and systematic sampling of the archipelago is necessary. Apart from Santa Cruz (in 1982, 1984) and Floreana (in 1997 and 2005) (Clark et al. 1982; Lubin 1984; Pezzatti et al. 1998; von Aesch 2006; HWH unpublished data), none of the other islands have been sampled extensively (Fig. 3). As a result, a multi-institutional project was initiated in 2020 to remedy this by surveying all islands. In addition, revision of taxonomic material in yet unexplored collections is underway. Revisions of these collections and systematic field surveys will provide the much-needed information to understand the role of ant species in ecosystem processes in the Galápagos as well as for prioritizing the management of introduced and invasive species and protecting endemic species.

## ﻿Checklist and identification keys

### ﻿Key to the subfamilies of the Galápagos Islands

**Table:** 

1	Mesosoma attached to the gaster by a single intermediate segment, the petiole (Fig. 44C)	**2**
–	Mesosoma attached to the gaster by two intermediate segments, the petiole, and post-petiole (Fig. 36C)	**Myrmicinae**
2	Gaster with a slight to remarkable constriction between its first and second segments (Fig. 45C); in the first case mandibles elongated (Fig. 48C); last segment of the gaster with sting, sometimes visible	**3**
–	Gaster without constriction between its first and second segments (Fig. 8D); never with long and slender mandibles; last segment of the gaster without sting	**4**
3	Pygidium with small spines or denticles (Fig. 7C); antennal scape short and robust, never surpassing the middle of the eyes; funiculus robust, with segments increasing progressively in size toward the apex; head in frontal view with frontal carinae very well marked, and with thick longitudinal ridges running from occipital margin towards the clypeus (Fig. 7D)	(**Dorylinae**) ***Cylindromyrmexwhymperi***
–	Pygidium without spines or denticles (Fig. 44D); antennal scape surpassing the middle of the eyes (long and slender); head in frontal view without ridges	**Ponerinae**
4	Apex of abdomen with a circular orifice surrounded by a fringe of short setae, the acidopore, formed from the hypopygium (Fig. 8E)	**Formicinae**
–	Apex of abdomen without acidopore (Fig. 4D)	**Dolichoderinae**

### ﻿Key to species and subspecies of the subfamily Dolichoderinae

**Table:** 

1	In lateral and dorsal views, petiole visible (Fig. 4B, D); cluster of long hairs located in the ventral surface of the head, the psammophore (Fig. 4E); dorsopropodeum with cone (Fig. 4D); head and mesosoma reddish brown, gaster, funiculus of antenna, petiole, and legs brownish black (Fig. 5B) *Dorymyrmex*)	***Dorymyrmexpyramicusalbemarlensis***
–	Petiole squamiform and notably reduced, in lateral and dorsal view, hidden under the first segment of the gaster (Fig. 5B, E); psammophore lacking on the ventral surface of the head; propodeum without cone on the dorsum (Fig. 5D) (*Tapinoma*)	**2**
2	Small (~ 1.5 mm), head mesosoma and antennae pale brown, legs and gaster pale yellow (Fig. 5A, B); anterior margin of clypeus relatively straight (Fig. 5C)	***Tapinomamelanocephalum***
–	Ants measuring ~ 2 mm with body and legs brown-gray (Fig. 6A, B); anterior base of clypeus slightly concave in the middle (Fig. 6C)	***Tapinoma* sp. hh07**

### ﻿Genus *Dorymyrmex* Mayr, 1866

***Dorymyrmexpyramicusalbemarlensis* Wheeler, 1919**

Fig. 4

**Remarks.** In Wheeler (1924) [CAS], Wheeler (1933). Cited as *Conomyrmapyramicaalbemarlensis* (Linsley and Usinger 1966), *Conomyrma* sp. (Clark et al. 1982) [ICCDRS], *C.pyramica* (Lubin 1983), *C.albemarlensis*, *C.pyramica* (Lubin 1984), *C.albemarlensis* (Lubin 1985), *Conomyrma* sp. (Meier 1994), *Dorymyrmexpyramicus* (Abedrabbo 1994) [ICCDRS] and *C.albemarlensis* (de la Vega 1994). Registered also in Roque-Albelo et al. (2000) [ICCDRS], Herrera and Causton (2010) [ICCDRS], Herrera (2015), Herrera (2019) and Herrera et al. (2020) [ICCDRS, RBINS].

**Figure 4.**
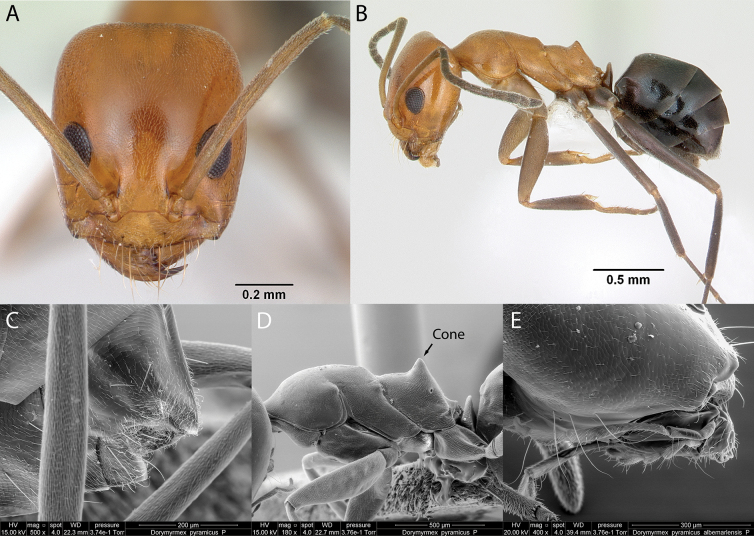
*Dorymyrmexpyramicusalbemarlensis* worker micrographs in **A** head in full-face view **B** view in profile, and SEM images of **C** apex of abdomen **D** mesosoma in profile **E** maxillary and labial palps.

**Taxonomic history**. Kempf (1972), Bolton (1995, 2014), Bolton et al. (2006).

**Distribution**. Possibly endemic: Baltra, Bartolomé, Daphne Mayor, Edén, Española, Fernandina, Genovesa, Isabela (VA, VD, VW), Marchena, Pinta, Rábida, Santa Cruz, Santa Fé, Santiago (Herrera et al. 2020).

### ﻿Genus *Tapinoma* Foerster, 1850

***Tapinomamelanocephalum* (Fabricius, 1793)**

Fig. 5

**Remarks.** Originally described as *Formicamelanocephalum* (Fabricius, 1793). First published record Emery (1893), cited also in Wheeler (1919) [CAS], Wheeler (1924), Linsley and Usinger (1966), Clark et al. (1982) [ICCDRS], Lubin (1984) [ICCDRS], [Bibr B53][Bibr B54], 1993), Abedrabbo (1994) [ICCDRS], Brandão and Paiva (1994), de la Vega (1994), Meier (1994) [ICCDRS], Peck et al. (1998), Pezzatti et al. (1998) [ICCDRS], Roque-Albelo et al. (2000) [ICCDRS], von Aesch and Cherix (2005) [ICCDRS], Boada (2005) [ICCDRS], von Aesch (2006) [ICCDRS], Causton et al. (2006), McMullen (2009), Herrera and Causton (2010) [ICCDRS], McMullen (2012), Chamorro et al. (2012) [ICCDRS], Dekoninck et al. (2014) [ICCDRS, RBINS], Wauters et al. (2016) [ICCDRS, RBINS], Herrera (2015, 2019), and Herrera et al. (2020) [ICCDRS, RBINS].

**Figure 5.**
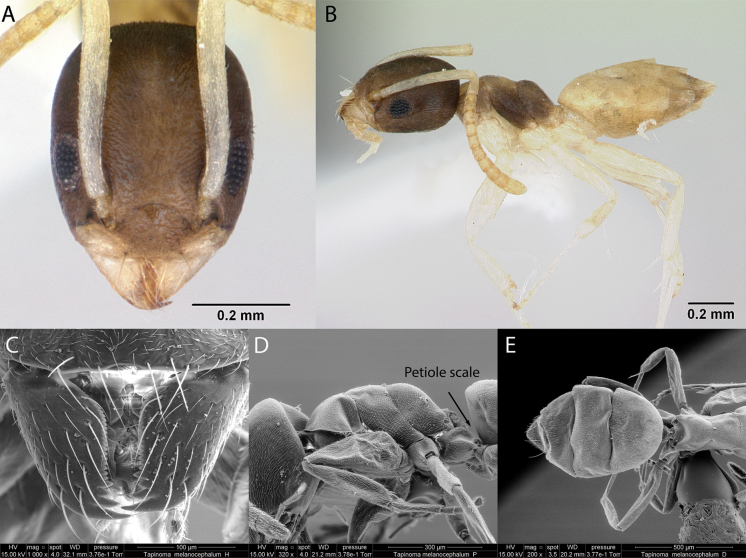
*Tapinomamelanocephalum* worker micrographs in **A** head in full-face view **B** view in profile, and SEM images of **C** close-up mandibles showing dentition **D** mesosoma in profile **E** gaster in dorsal view.

**Taxonomic history**. Kempf (1972), Bolton (1995, 2014), Bolton et al. (2006).

**Distribution**. Afrotropical, Australasia, Indomalaya, Malagasy, Nearctic, Neotropical, Oceania, Palearctic.

**Galápagos distribution**. Introduced: Albany, Baltra, Champion, Española, Fernandina, Floreana, Genovesa, Isabela (CA, SN, VA), Marchena, Pinta, Plaza Sur, Rábida, Santiago, San Cristóbal, Santa Cruz, Seymour Norte, Santa Fé (Herrera et al. 2020).

**New record**. Mariela Mediana Islet.

***Tapinoma* sp. hh07**

Fig. 6

**Remarks.** In Herrera et al. (2014) [ICCDRS], Herrera (2015), and Herrera et al. (2020) [ICCDRS].

**Figure 6.**
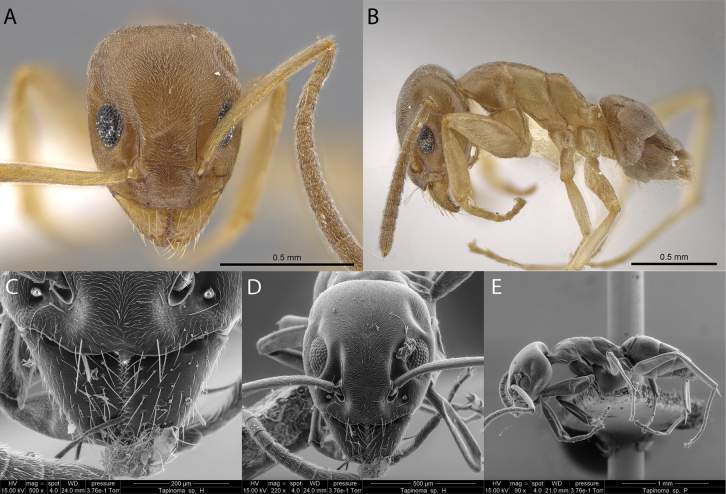
*Tapinoma* sp. hh07 worker micrographs in **A** head in full-face view **B** view in profile, and SEM images of **C** close-up mandibles showing dentition **D** head in full-face view **E** view in profile.

**Distribution.** Undetermined origin: Santa Cruz (Herrera et al. 2014).

### ﻿Genus *Cylindromyrmex* Mayr, 1870

***Cylindromyrmexwhymperi* (Cameron, 1891)**

Fig. 7

**Remarks.** Originally described as *Holcoponerawhymperi* (Cameron, 1891). Cited as *Cylindromyrmexstriatus* in Wheeler (1919) [CAS]. *Cylindromyrmexwilliamsi* in Wheeler (1924). *Cylindromyrmexstriatustibialis* in Stitz (1932). *Cylindromyrmexwilliamsi* in Linsley and Usinger (1966), *Cylindromyrmex* sp. in Silberglied (1972). *Cylindromyrmexstriatus* in Lubin (1984), *Cylindromyrmex* sp. in de la Vega (1994). *Cylindromyrmexwhymperi* in De Andrade (1998), *Cylindromyrmexstriatus* in Causton et al. (2006), *Cylindromyrmexwhymperi*Herrera and Causton (2010) [ICCDRS], Herrera (2015, 2019) and Herrera et al. (2020) [ICCDRS].

**Figure 7.**
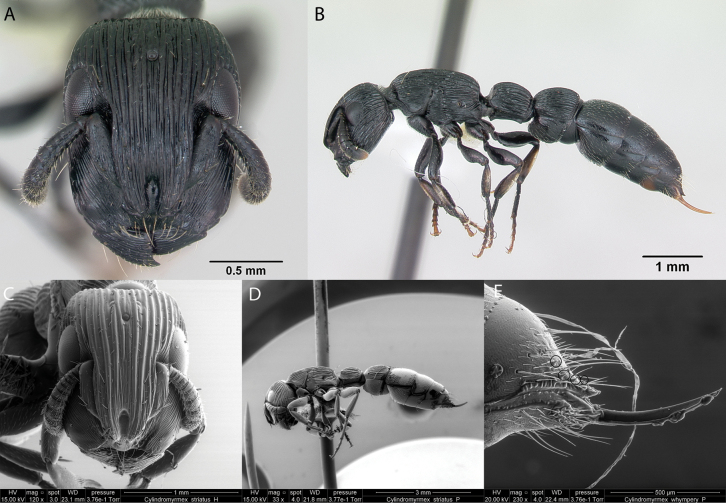
*Cylindromyrmexwhymperi* worker micrographs in **A** head in full-face view **B** view in profile, and SEM images of **C** head in full-face view **D** view in profile **E** stinging apparatus. The small circles indicate spines on the pygidium.

**Taxonomic history**. Kempf (1972), Bolton (1995, 2014), De Andrade (1998), Bolton et al. (2006).

**Distribution**. Neotropical.

**Galápagos distribution**. Introduced: Baltra, Fernandina, Isabela (VA, VW), Santa Cruz (Herrera and al. 2020).

**New record**. Santiago Island.

### ﻿Key to the genera and species of the subfamily Formicinae

**Table:** 

1	Antenna, including scape, with 9 segments (Fig. 8C) (*Brachymyrmex*)	***Brachymyrmexheeri***
–	Antenna, including scape, with > 9 segments (Fig. 11C)	**2**
2	Polymorphic, minor workers > 4 mm (total length); antennal insertions located distantly from posterior margin of the clypeus (Fig. 11D); head in dorsal view with frontal carinae (Fig. 11D) (*Camponotus*)	**3**
–	Monomorphic, workers of small size, < 4 mm (total length), with antennal insertions located near to posterior margin of clypeus (Fig. 12A, C); head in dorsal view with frontal carinae hardly visible (Fig. 11C)	**5**
3	In lateral view, promesonotum and dorsum of propodeum flat; propodeal declivity angulate (Fig. 11E); short and erect hairs distributed evenly along mesosoma; head, mesosoma, and gaster black with antennae and legs reddish (Fig. 11A, B)	***Camponotusplanus***
–	In lateral view, promesonotum and propodeum rounded until the base of the declivity of propodeum, forming a single convexity (Figs 9B, E, 10B, C); long and erect hairs distributed unevenly along mesosoma; ants yellowish (Figs 9B, 10B)	**4**
4	Longitudinal carina visible in middle of the clypeus (major workers); head in frontal view with frontal carinae closing towards the middle of eyes; mesosoma with > 10 erect hairs (Fig. 9B, E)	***Camponotuszonatus***
–	Longitudinal carina in the middle of clypeus inconspicuous or absent (major workers); head in frontal view with frontal carinae opening from base of fronto-clypeal suture towards middle of eyes; mesosoma with < 10 erect hairs (Fig. 10B, C)	***Camponotusmacilentus***
5	Scape obviously elongate without erect setae and extending at least twice the length of the head in lateral view (Fig. 13D); mandibles with 5 teeth; mesosoma smooth with absence of appressed hairs (Fig. 13B, E) (*Paratrechina*)	***Paratrechinalongicornis***
–	Scape with abundant erect setae and never extending twice the length of the head in lateral view (Fig. 12C, D); mandible with 6 or 7 teeth; mesosoma with appressed hairs (Fig. 12E) (*Nylanderia*)	**6**
6	Head, mesosoma, gaster and legs dark brown with trochanters yellowish; mesopleuron and metapleuron smooth and shiny (Fig. 12A, B)	***Nylanderiasteinheili***
–	Species without combination of characteristics described above	***Nylanderia* spp**.

### ﻿Genus *Brachymyrmex* Mayr, 1868

***Brachymyrmexheeri* Forel, 1874**

Fig. 8

**Remarks.** First published record in Herrera and Longino (2008) [ICCDRS]. Cited as *Brachymyrmex* sp. in Causton et al. (2006). *Brachymyrmexheeri* in Dekoninck et al. (2014) [ICCDRS, RBINS], Wauters et al. (2014) [ICCDRS], Wauters et al. (2016) [ICCDRS], Herrera (2015, 2019) and Herrera et al. (2020) [ICCDRS].

**Figure 8.**
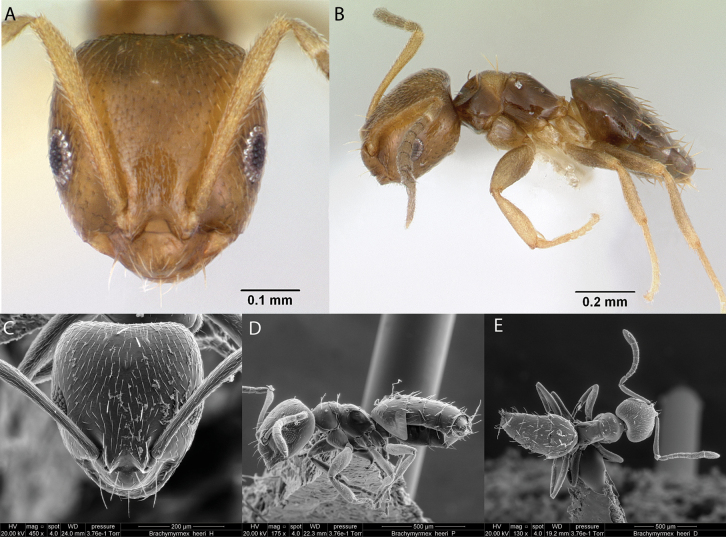
*Brachymyrmexheeri* worker micrographs in **A** head in full-face view **B** view in profile, and SEM images of **C** head in full-face view **D** view in profile **E** head and antenna in dorsal view.

**Taxonomic history**. Bolton (1995, 2014), Bolton et al. (2006).

**Distribution.** Nearctic, Neotropical, and Palearctic.

**Galápagos distribution**. Introduced: Floreana, Isabela (SN, VE), Marchena, San Cristóbal, Santa Cruz (Herrera et al. 2020).

### ﻿Genus *Camponotus* Mayr, 1861

***Camponotuszonatus* Emery, 1894**

Fig. 9

**Remarks.** First published record (Herrera and Causton 2010) [ICCDRS]. Cited in Dekoninck et al. (2014) [ICCDRS, RBINS], Wauters et al. (2016) [RBINS], Herrera (2015, 2019), and Herrera et al. (2020) [ICCDRS, RBINS].

**Figure 9.**
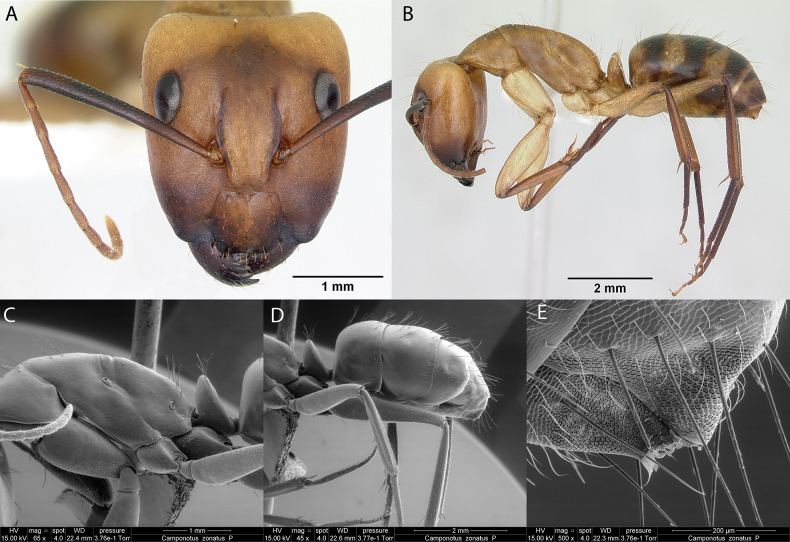
*Camponotuszonatus* worker micrographs in **A** head in full-face view **B** view in profile, and SEM images of **C** mesosoma in profile **D** gaster in profile view **E** close up of acidopore.

**Taxonomic history**. Bolton (1995, 2014), Bolton et al. (2006).

**Distribution.** Neotropical.

**Galápagos distribution**. Introduced: Bainbridge #1, Bainbridge #3, Bainbridge #4, Bainbridge #5, Bainbridge #6, Baltra, Champion, Cuevas, Daphne Mayor, Eden, Floreana, Genovesa, Isabela (CA, SN, VA, VD, VW), Mao, Marchena, Pinta, Pinzón, Plaza Norte, Plaza Sur, San Cristóbal, Santa Cruz, Santa Fé, Santiago, Seymour Norte (Herrera et al. 2020).

***Camponotusmacilentus* Smith, 1877**

Fig. 10

**Remarks.** Cited as Camponotus (Myrmamblys) macilentus in Wheeler (1919). Camponotus (Pseudocolobopsis) macilentus in Emery (1920). Camponotus (Pseudocolobopsis) macilentus macilentus in Linsley and Usinger (1966). Camponotus (Pseudocolobopsis) macilentus in Kempf (1972). *Camponotusmacilentus* in Clark et al. (1982), Lubin (1983) [ICCDRS], Lubin (1984, 1985), Brandão and Paiva (1994), Meier (1994), Peck (1994b), Bolton (1995), Roque-Albelo et al. (2000) [ICCDRS], Boada (2005) [ICCDRS], McMullen (2011, 2012). Misidentification in Pezzatti et al. (1998) [ICCDRS], von-Aesch and Cherix (2005), von Aesch (2006) [ICCDRS], Herrera (2015, 2019) and Herrera et al. (2020) [ICCDRS, RBINS].

**Figure 10.**
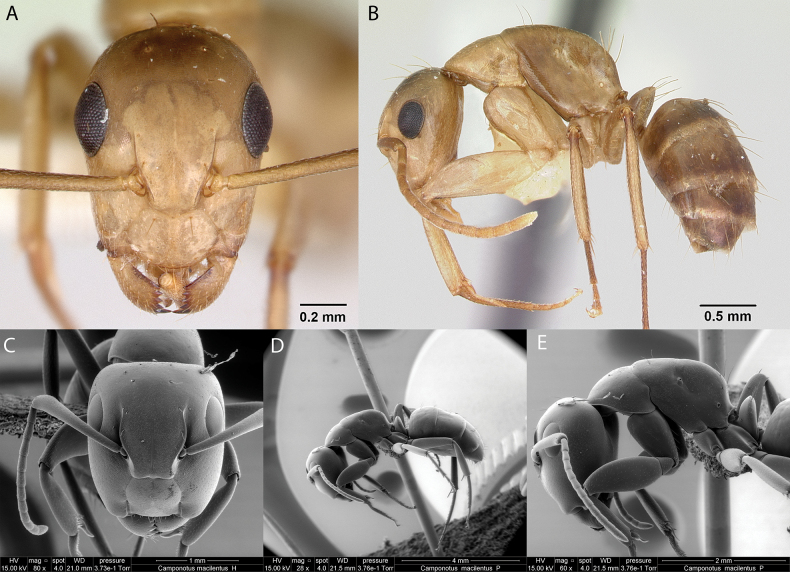
*Camponotusmacilentus* worker micrographs in **A** head in full-face view **B** view in profile, and SEM images of **C** head in full-face view **D** view in profile **E**. mesosoma and head profile view.

**Taxonomic history.**Bolton (1995, 2014), Bolton et al. (2006).

**Distribution.** Endemic: Baltra, Champion, Española, Fernandina, Floreana, Genovesa, Isabela (SN, VA, VD, VW), Marchena, Pinta, Pinzón, Plaza Norte, Rábida, Santa Cruz, Santa Fé, Santiago, (Herrera and al. 2020).

**Citations.**

***Camponotusmacilentusalbemarlensis* Wheeler**, **1919**. Cited as Camponotus (Myrmamblys) macilentus var. albemarlensis Wheeler, 1919: 284. Camponotus (Pseudocolobopsis) macilentus var. albemarlensis in Emery (1925). Camponotus (Pseudocolobopsis) macilentus albemarlensis in Linsley and Usinger (1966). Camponotus (Pseudocolobopsis) macilentus var. albemarlensis in Kempf (1972). Camponotus (Pseudocolobopsis) macilentus albemarlensis in (Bolton, 1995), Herrera (2015, 2019).

**Taxonomic history.**Bolton (1995, 2014), Bolton et al. (2006).

**Distribution.** Endemic: Isabela Island.

***Camponotusmacilentusaltinotus* Stitz**, **1932**. Cited as Camponotus (Pseudocolobopsis) macilentus var. altinota Stitz, 1932: 370. Camponotus (Pseudocolobopsis) macilentus altinotus in Linsley and Usinger (1966). Camponotusmacilentusvar.altinotus in Kempf (1972). Camponotus (Pseudocolobopsis) macilentus altinotus in (Bolton, 1995), Herrera (2015, 2019).

**Taxonomic history.**Bolton (1995, 2014), Bolton et al. (2006).

**Distribution.** Endemic: Floreana Island (Stitz 1932).

***Camponotusmacilentusbarringtonensis* Wheeler**, **1919**. Cited as Camponotus (Myrmamblys) macilentus var. barringtonensis Wheeler, 1919: 282. Camponotus (Pseudocolobopsis) macilentus var. barringtonensis in Emery (1925). Camponotus (Pseudocolobopsis) macilentus barringtonensis in Linsley and Usinger (1966). Camponotus (Pseudocolobopsis) macilentus var. barringtonensis in Kempf (1972). Camponotus (Pseudocolobopsis) macilentus barringtonensis in (Bolton, 1995), Herrera (2015, 2019).

**Taxonomic history.**Bolton (1995, 2014), Bolton et al. (2006).

**Distribution.** Endemic: Santa Fé Island (Wheeler 1919).

***Camponotusmacilentusbindloensis* Wheeler**, **1919**. Cited as Camponotus (Myrmamblys) macilentus var. bindloensis Wheeler, 1919: 286. Camponotus (Pseudocolobopsis) macilentus var. bindloensis in Emery (1925). Camponotus (Pseudocolobopsis) macilentus bindloensis in Linsley and Usinger (1966). Camponotus (Pseudocolobopsis) macilentus var. bindloensis in Kempf (1972), Camponotus (Pseudocolobopsis) macilentus bindloensis in Bolton (1995). *Camponotusmacilentusbindloensis* in Herrera (2015, 2019).

**Taxonomic history.**Bolton et al. (2006), Bolton (2014).

**Distribution.** Endemic: Marchena Island (Wheeler 1919).

***Camponotusmacilentuscastellanus* Wheeler**, **1924**. Cited as Camponotus (Myrmamblys) macilentus var. castellanus Wheeler, 1924: 116. Cited as Camponotus (Pseudocolobopsis) macilentus castellanus in Linsley and Usinger (1966). Camponotus (Pseudocolobopsis) macilentus var. castellanus in Kempf (1972). Camponotus (Pseudocolobopsis) macilentus castellanusBolton (1995), Herrera (2015, 2019).

**Taxonomic history.**Bolton (1995, 2014), Bolton et al. (2006).

**Distribution.** Endemic: Genovesa Island (Wheeler 1924).

***Camponotusmacilentusduncanensis* Wheeler**, **1919**. Cited as Camponotus (Myrmamblys) macilentus var. duncanensis Wheeler, 1919: 283. Camponotus (Pseudocolobopsis) macilentus var. duncanensis in Emery (1925). Camponotus (Pseudocolobopsis) macilentus duncanensis in Linsley and Usinger (1966). Camponotus (Pseudocolobopsis) macilentus var. duncanensis in Kempf (1972). *Camponotusmacilentusduncanensis* in Bolton (1995), Herrera (2015, 2019).

**Taxonomic history.**Bolton (1995, 2014), Bolton et al. (2006).

**Distribution.** Endemic: Floreana, Pinzón Islands (Wheeler 1919, Stitz 1932).

***Camponotusmacilentushoodensis* Wheeler**, **1919**. Cited as Camponotus (Myrmamblys) macilentus var. hoodensis Wheeler, 1919: 285. Cited ad Camponotus (Pseudocolobopsis) macilentus var. hoodensis in Emery (1925). Camponotus (Pseudocolobopsis) macilentus hoodensis in Linsley and Usinger (1966). Camponotus (Pseudocolobopsis) macilentus var. hoodensis in Kempf (1972). Camponotus (Pseudocolobopsis) macilentus hoodensis in Bolton (1995), Herrera (2015, 2019).

**Taxonomic history.**Bolton (1995, 2014), Bolton et al. (2006).

**Distribution.** Endemic: Española Island (Wheeler 1919).

***Camponotusmacilentusjacobensis* Wheeler**, **1919**. Cited as Camponotus (Myrmamblys) macilentus var. jacobensis Wheeler, 1919: 280. Camponotus (Pseudocolobopsis) macilentus var. jacobensis in Emery (1925). Camponotus (Pseudocolobopsis) macilentus jacobensis in Linsley and Usinger (1966). Camponotus (Pseudocolobopsis) macilentus var. jacobensis in Kempf (1972). Camponotus (Pseudocolobopsis) macilentus jacobensis in Bolton (1995), Herrera (2015, 2019).

**Taxonomic history.**Bolton (1995, 2014), Bolton et al. (2006).

**Distribution.** Endemic: Santiago Island (Wheeler 1919).

***Camponotusmacilentusnarboroensis* Wheeler**, **1919**. Cited as Camponotus (Myrmamblys) macilentus var. narboroensis Wheeler, 1919: 286. Camponotus (Pseudocolobopsis) macilentus var. narboroensis in Emery (1925). Camponotus (Pseudocolobopsis) macilentus narboroensis in Linsley and Usinger (1966). Camponotus (Pseudocolobopsis) macilentus var. narboroensis in Kempf (1972). Camponotus (Pseudocolobopsis) macilentus narboroensis in Bolton (1995), Herrera (2015, 2019).

**Taxonomic history.**Bolton (1995, 2014), Bolton et al. (2006).

**Distribution.** Endemic: Fernandina Island (Wheeler 1919, 1933).

***Camponotusmacilentuspervicus* Wheeler**, **1924**. Cited as Camponotus (Myrmamblys) macilentus var. pervicus Wheeler, 1924: 115. Camponotus (Pseudocolobopsis) macilentus pervicus in Linsley and Usinger (1966). Camponotus (Pseudocolobopsis) macilentus var. pervicus in Kempf (1972). Camponotus (Pseudocolobopsis) macilentus pervicus in Bolton (1995), Herrera (2015, 2019).

**Taxonomic history.**Bolton (1995, 2014), Bolton et al. (2006).

**Distribution.** Endemic: Santa Cruz Island (Wheeler 1924).

***Camponotusmacilentussapphirinus* Wheeler**, **1924**. Cited as Camponotus (Myrmamblys) macilentus var. sapphirinus Wheeler, 1924: 114. Cited as Camponotus (Pseudocolobopsis) macilentus sapphirinus in Linsley and Usinger (1966). Camponotus (Pseudocolobopsis) macilentus var. sapphirinus in Kempf (1972). Camponotus (Pseudocolobopsis) macilentus sapphirinus in Bolton (1995), Herrera (2015).

**Taxonomic history.**Bolton (1995, 2014), Bolton et al. (2006).

**Distribution.** Endemic: Santa Cruz, Baltra Islands (Wheeler 1924).

***Camponotusmacilentusvulcanalis* Wheeler**, **1919**. Cited as Camponotus (Myrmamblys) macilentus var. vulcanalis Wheeler, 1919: 284. Camponotus (Pseudocolobopsis) macilentus var. vulcanalis in Emery (1925). Camponotus (Pseudocolobopsis) macilentus vulcanalis in Linsley and Usinger (1966). Camponotus (Pseudocolobopsis) macilentus var. vulcanalis in Kempf (1972). Camponotus (Pseudocolobopsis) macilentus vulcanalis in Bolton (1995), Herrera (2015, 2019).

**Taxonomic history.**Bolton (1995, 2014), Bolton et al. (2006).

**Distribution.** Endemic: Isabela Island (Wheeler 1919).

***Camponotusmacilentuswollebaeki* Stitz**, **1932**. Cited as Camponotus (Myrmamblys) macilentus var. wollebaeki Stitz, 1932: 371. Camponotus (Pseudocolobopsis) macilentus wollebaeki in Linsley and Usinger (1966). Camponotusmacilentusvar.wollebaeki in Kempf (1972). Camponotus (Pseudocolobopsis) macilentus wollebaeki in Bolton (1995), Herrera (2015, 2019).

**Taxonomic history.**Bolton (1995, 2014), Bolton et al. (2006).

**Distribution.** Endemic: Floreana Island (Stitz 1932).

***Camponotusplanus* Smith, 1877**

Fig. 11

**Remarks.** Cited as Camponotus (Myrmorhachis) planus in Wheeler (1919), Emery (1920). Camponotus (Myrmocladoecus) planus in Wheeler (1924), Stitz (1932). Camponotus (Myrmocladoecus) planus planus in Linsley and Usinger (1966). Camponotus (Myrmocladoecus) planus in Kempf (1972). *Camponotusplanus* in Clark et al. (1982) [ICCDRS], Lubin (1983, 1984, 1985) [ICCDRS], McMullen (1993), Brandão and Paiva (1994), de la Vega (1994), Meier (1994) [ICCDRS]. Camponotus (Myrmocladoecus) planus in Bolton (1995). *Camponotusplanus* in Pezzatti et al. (1998) [ICCDRS], Roque-Albelo et al. (2000) [ICCDRS], von Aesch and Cherix (2005) [ICCDRS], Boada (2005) [ICCDRS], von Aesch (2006) [ICCDRS], Jaramillo et al. (2010), Herrera and Causton (2010) [ICCDRS], Chamorro et al. (2012) [ICCDRS], Herrera (2015, 2019) [ICCDRS] and Wauters (2016) [ICCDRS; RBINS].

**Figure 11.**
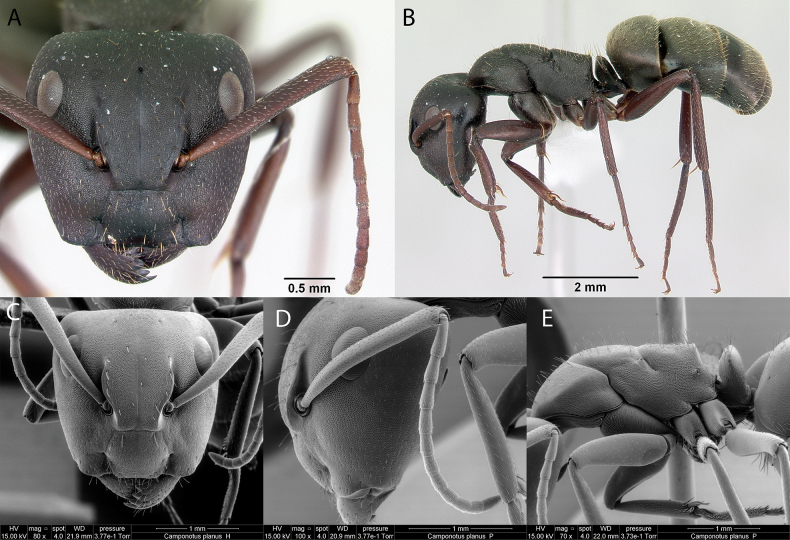
*Camponotusplanus* worker micrographs in **A** head in full-face view **B** view in profile, and SEM images of **C** head in full-face view **D** head profile view **E** mesosoma in profile.

**Taxonomic history.**Kempf (1972), Bolton (1995, 2014), Bolton et al. (2006).

**Distribution.** Endemic: Bainbridge #1, Baltra, Bartolomé, Cousin, Fernandina, Floreana, Isabela (CA, SN, VA, VD, VE, VW), Logie, Marchena, Pinzón, Plaza Sur, Rábida, Santiago, San Cristóbal, Santa Cruz, Seymour Norte, Santa Fé (Herrera et al. 2020).

**Citations.**

***Camponotusplanusfernandinensis* Wheeler**, **1919**. Cited as Camponotus (Myrmorhachis) planus var. fernandinensis Wheeler, 1919: 296. Camponotus (Myrmocladoecus) planus fernandinensis in Linsley and Usinger (1966). Camponotus (Myrmocladoecus) planus var. fernandinensis in Kempf (1972). Camponotus (Myrmocladoecus) planus fernandinensis in Bolton (1995), Herrera (2015, 2019).

**Taxonomic history.**Bolton (1995, 2014), Bolton et al. (2006).

**Distribution.** Endemic: Fernandina Island (Wheeler 1919).

***Camponotusplanusfidelis* Wheeler**, **1919**. Cited as Camponotus (Myrmorhachis) planus var. fidelis Wheeler, 1919: 295. Camponotus (Myrmocladoecus) planus var. fidelis in Emery (1925). Camponotus (Myrmocladoecus) planus fidelis in Linsley and Usinger (1966). Camponotus (Myrmocladoecus) planus var. fidelis in Kempf (1972). Camponotus (Myrmocladoecus) planus fidelis in Bolton (1995), Herrera (2015, 2019).

**Taxonomic history.**Bolton (1995, 2014), Bolton et al. (2006).

**Distribution.** Endemic: Santa Fé (Wheeler 1919).

***Camponotusplanushephaestus* Wheeler**, **1933**. Cited as Camponotus (Myrmorhachis) planus var. hephaestus Wheeler, 1933: 59. Camponotus (Myrmocladoecus) planus hephaestus in Linsley and Usinger (1966). Camponotus (Myrmocladoecus) planus var. hephaestus in Kempf (1972). Camponotus (Myrmocladoecus) planus hephaestus in Bolton (1995), Herrera (2015, 2019).

**Taxonomic history.**Bolton (1995, 2014), Bolton et al. (2006).

**Distribution.** Endemic: Isabela Island (Wheeler 1933).

***Camponotusplanusindefessus* Wheeler**, **1919**. Cited as Camponotus (Myrmorhachis) planus var. indefessus Wheeler, 1919: 294. Camponotus (Myrmocladoecus) planus indefessus in Linsley and Usinger (1966). Camponotus (Myrmocladoecus) planus var. indefessus in Kempf (1972). Camponotus (Myrmocladoecus) planus indefessus in Bolton (1995), Herrera (2015, 2019).

**Taxonomic history.**Bolton (1995, 2014), Bolton et al. (2006).

**Distribution.** Endemic: Santa Cruz Island (Wheeler 1919).

***Camponotusplanusisabelensis* Wheeler**, **1919**. Cited as Camponotus (Myrmorhachis) planus var. isabelensis Wheeler, 1919: 293. Camponotus (Myrmocladoecus) planus isabelensis in Linsley and Usinger (1966). Camponotus (Myrmocladoecus) planus var. isabelensis in Kempf (1972). Camponotus (Myrmocladoecus) planus isabelensis in Bolton (1995), Herrera (2015, 2019).

**Taxonomic history.**Bolton (1995, 2014), Bolton et al. (2006).

**Distribution.** Endemic: Isabela Island (Wheeler 1919, 1933).

***Camponotusplanusperegrinus* Emery**, **1893**. Cited as *Camponotusperegrinus* Emery, 1893: 91. Camponotus (Myrmorhachis) planus peregrinus in Wheeler (1919). Camponotus (Myrmocladoecus) planus var. peregrinus in Wheeler (1924), Stitz (1932). Camponotus (Myrmocladoecus) planus peregrinus in Linsley and Usinger (1966). Camponotus (Myrmocladoecus) planus var. peregrinus in Kempf (1972). Camponotus (Myrmocladoecus) planus peregrinus in Bolton (1995), Herrera (2015, 2019).

**Taxonomic history.**Bolton (1995, 2014), Bolton et al. (2006).

**Distribution.** Endemic: Floreana, San Cristóbal Island (Wheeler 1919; Stitz 1932; Wheeler 1933).

***Camponotusplanuspinzonensis* Wheeler**, **1919**. Cited as Camponotus (Myrmorhachis) planus var. pinzonensis Wheeler, 1919: 297. Camponotus (Myrmocladoecus) planus var. pinzonensis in Emery (1925). Camponotus (Myrmocladoecus) planus pinzonensis in Linsley and Usinger (1966). Camponotus (Myrmocladoecus) planus var. pinzonensis in Kempf (1972). Camponotus (Myrmocladoecus) planus pinzonensis in Bolton (1995), Herrera (2015, 2019).

**Taxonomic history.**Bolton (1995, 2014), Bolton et al. (2006).

**Distribution.** Endemic: Pinzón Island (Wheeler 1919).

***Camponotusplanussansalvadorensis* Wheeler**, **1924**. Cited as Camponotus (Myrmorhachis) planus var. sansalvadorensis Wheeler, 1924: 119. Cited as Camponotus (Myrmocladoecus) planus sansalvadorensis in Linsley and Usinger (1966). Camponotus (Myrmocladoecus) planus var. sansalvadorensis in Kempf (1972). Camponotus (Myrmocladoecus) planus sansalvadorensis in Bolton (1995), Herrera (2015, 2019).

**Taxonomic history.**Bolton (1995, 2014), Bolton et al. (2006).

**Distribution.** Endemic: Santiago Island (Wheeler 1924).

***Camponotusplanussantacruzensis* Wheeler**, **1919**. Cited as Camponotus (Myrmorhachis) planus var. santacruzensis Wheeler, 1919: 294. Camponotus (Myrmocladoecus) planus var. santacruzensis in Wheeler (1924). Camponotus (Myrmocladoecus) planus santacruzensis in Linsley and Usinger (1966). Camponotus (Myrmocladoecus) planus var. santacruzensis in Kempf (1972). Camponotus (Myrmocladoecus) planus santacruzensis in Bolton (1995), Herrera (2015, 2019).

**Taxonomic history.**Bolton (1995, 2014), Bolton et al. (2006).

**Distribution.** Endemic: Santa Cruz, Baltra Island (Wheeler 1919, 1924, 1933).

***Camponotussenex* (Smith**, **1858).** Originally described as *Formicasenex* (Smith, 1858). Cited in Smith (1877), Wheeler (1919). Doubtful record for Galápagos (Wheeler 1924). Cited also in Linsley and Usinger (1966), Kempf (1972), Brandão and Paiva (1994) and Herrera (2015, 2019).

**Taxonomic history.**Bolton (1995, 2014), Bolton et al. (2006).

**Distribution.** Neotropical.

**Galápagos distribution.** Uncertain: San Cristóbal Island (Smith 1877).

### ﻿Genus *Nylanderia* Emery, 1906

***Nylanderiafulvanesiotis* (Wheeler**, **1919)**

**Remarks.** Cited as *Prenolepisfulvanesiotis* in Wheeler (1919, 1924, 1933) [CAS]. As *Paratrechinafulvanesiotis* in Linsley and Usinger (1966) and *Nylanderiafulvanesiotis* in Kempf (1972), *Paratrechinanesiotis* in Lubin (1983, 1984, 1985), see also Herrera (2015, 2019).

**Taxonomic history.**Bolton (1995, 2014), Bolton et al. (2006).

**Distribution.** Neotropical.

**Galápagos distribution.** Introduced: Española, Isabela, Santiago, San Cristobal, Santa Cruz (Wheeler 1919, 1924, 1933).

***Nylanderiaguatemalensisitinerans* (Forel**, **1901)**

**Remarks.** Cited as *Prenolepisvividulaguatemalensisitinerans* in Wheeler (1919, 1924), Nylanderiavividulaguatemalensisvar.itinerans in Wheeler (1933) [CAS], *Paratrechinavividulaitinerans* in Linsley and Usinger (1966), Nylanderiaguatemalensisvar.itinerans in Kempf (1972), *Paratrechinavividulaitinerans* in Brandão and Paiva (1994) and *Paratrechinaguatemalensisitinerans* in Pezzatti et al. (1998). See also Herrera (2015, 2019).

**Taxonomic history.**Bolton (1995, 2014), Bolton et al. (2006).

**Distribution.** Neotropical.

**Galápagos distribution.** Introduced: Floreana, San Cristobal, Santa Cruz (Wheeler 1919, 1924, 1933).

***Nylanderiasteinheili* (Forel, 1893)**

Fig. 12

**Remarks.** Cited as *Prenolepissteinheili* in (Forel, 1893). First record in Herrera et al. (2014), cited also in Dekoninck et al. (2014), Herrera (2015a. b), Wauters et al. (2016) [ICCDRS].

**Figure 12.**
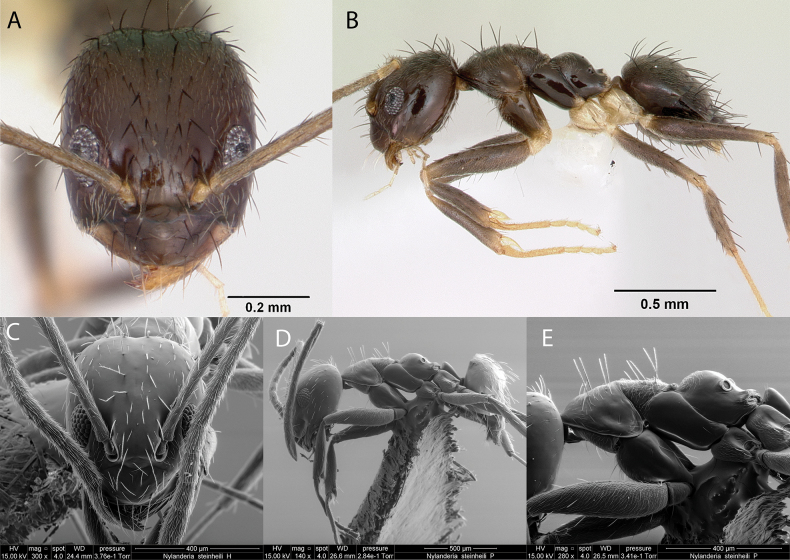
*Nylanderiasteinheili* worker micrographs in **A** head in full-face view **B** view in profile, and SEM images of **C** head in full-face view **D** view in profile **E** mesosoma in profile.

**Taxonomic history.**Kempf (1972), Brandão (1991), Bolton (1995, 2014), Bolton et al. (2006).

**Distribution.** Malagasy, Nearctic, Neotropical.

**Galápagos distribution.** Introduced: Floreana, Gardner (next to Floreana), Isabela (CA), Pinzón, San Cristóbal, Santa Cruz (Herrera et al. 2014, 2020) [ICCDRS].

**New record.** Santiago Island.

***Nylanderiavaga* (Forel**, **1901)**

**Remarks.** Cited as *Prenolepisvaga* in (Forel, 1901). Cited as *Paratrechinavaga* in Clark et al. (1982), McMullen (1987), McMullen (1990), McMullen (1993), Causton et al. (2006) and McMullen (2007). Cited as possibly *N.vaga* in Pezzatti et al. (1998).

**Taxonomic history.**Bolton (1995, 2014), Bolton et al. (2006).

**Distribution.** Australasia, Indomalaya, Neotropical, Oceania.

**Galápagos distribution.** Introduced: Floreana, Santa Cruz, Pinta (Clark et al. 1982; [Bibr B53][Bibr B54], 1993, 2007).

### ﻿Genus *Paratrechina* Motschoulsky, 1863

***Paratrechinalongicornis* (Latreille, 1802)**

Fig. 13

**Remarks.** Cited as *Formicalongicornis* in (Latreille, 1802). Cited as *Prenolepislongicornis* (Latreille, 1802) in Wheeler (1919), Wheeler (1924) and Stitz (1932). *Paratrechinalongicornis* in Wheeler (1933) [CAS], Kempf (1972), Linsley and Usinger (1966), Lubin (1984) [ICCDRS], McMullen (1987), McMullen (1990), McMullen (1993), Brandão and Paiva (1994), Meier (1994) [ICCDRS], Pezzatti et al. (1998) [ICCDRS], von Aesch and Cherix (2005) [ICCDRS], Causton et al. (2006) [ICCDRS], von Aesch (2006) [ICCDRS]. Also, in Herrera and Causton (2010) [ICCDRS]. Dekoninck et al. (2014) [ICCDRS, RBINS], Wauters et al. (2016) [RBINS], Herrera (2015, 2019) and Herrera et al. (2020) [ICCDRS, RBINS].

**Figure 13.**
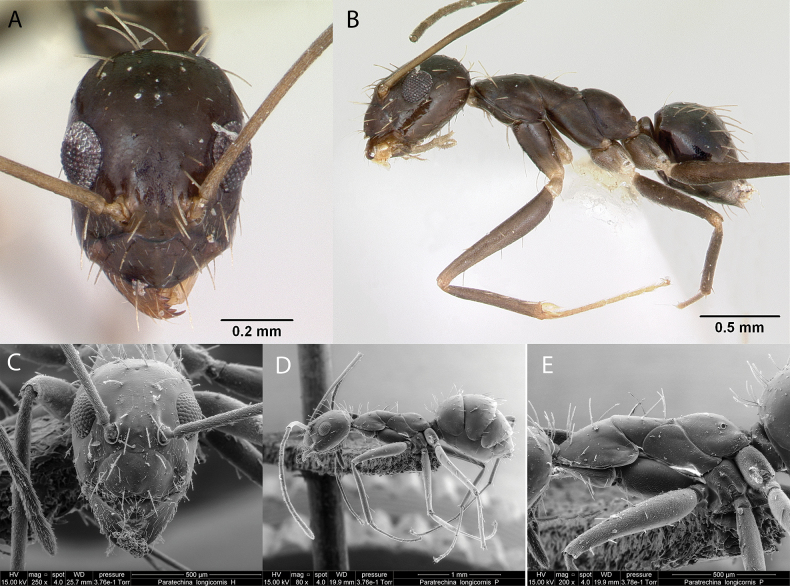
*Paratrechinalongicornis* worker micrographs in **A** head in full-face view **B** view in profile, and SEM images of **C** head in full-face view **D** view in profile **E** mesosoma in profile.

**Taxonomic history.**Bolton (1995, 2014), Bolton et al. (2006).

**Distribution.** Afrotropical, Australasia, Indomalaya, Malagasy, Nearctic, Neotropical, Oceania, Palearctic.

**Galápagos distribution.** Introduced: Baltra, Bartolomé, Champion, Española, Fernandina, Floreana, Gardner (next to Española), Isabela (SN, VA), Marchena, Pinta, Rábida, Santiago, San Cristóbal, Santa Cruz, Santa Fé, Seymour Norte (Herrera et al. 2020).

### ﻿Key to the genera and species of the subfamily Myrmicinae

**Table:** 

1	Postpetiole attached to the dorsal surface of the first segment of the gaster (*Crematogaster*) (Fig. 17C)	***Crematogaster* JTL – 022**
–	Postpetiole attached on anterior surface of the first segment of the gaster (Figs 14C, 40C)	**2**
2	Antenna with 12 segments, scape included (Fig. 14D)	**3**
–	Antenna with < 12 segments: scape included (Figs 18C, 33C)	**19**
3	Antennal club of two segments (Fig. 14D); triangular mandible equipped with a tooth at the basal margin; median portion of clypeus bicarinate with 2 clypeal teeth in the anterior clypeal margin, pointing to the apical margin of the mandibles when these are almost closed (Fig. 14E). Eye composed of 3 ommatidia (Fig. 14F) (*Adelomyrmex*)	***Adelomyrmexlonginoi***
–	Antennal club diffuse or with 2 or 3 segments (Fig. 42A, C); mandible triangular with absence of teeth on the basal margin; pair of apical teeth at the anterior margin of clypeus absent (Fig. 42D)	**4**
4	Propodeum without spines (Figs 21C, 22F)	**5**
–	Propodeum armed with spines (Figs 16C, 38C, 37C)	**8**
5	Posterior surface of the head and propodeal dorsum transversely striate (Fig. 42E, F) (*Trichomyrmex*)	***Trichomyrmexdestructor***
–	Posterior surface of the head and propodeal dorsum not transversely striate (Figs 22D, 23D); (*Monomorium*)	**6**
6	Head, mesosoma and gaster smooth and shiny (Fig. 21A, B, D, E); in lateral view mesosoma with > 4 erect setae (Fig. 21C); bicolored with mesosoma pale brown, head and gaster dark brown (Fig. 21A, B)	***Monomoriumfloricola***
–	Head and mesosoma neither smooth nor shiny (Figs 22A, C, D, E, 23C, D, E); in lateral view mesosoma with ≤ 4erect setae (Fig. 22D), in dorsal and lateral view with appressed hairs (Fig. 23D, E)	**7**
7	In lateral or dorsal view, pronotum with a pair of erect setae (Fig. 22D, E), in lateral view post-petiole almost the same size as petiole (Fig. 22B, F); ant yellowish in its entirety (Fig. 22B)	***Monomoriumpharaonis***
–	In lateral or dorsal view, pronotum without a pair of erect setae, only appressed pubescence present (Fig. 23E, D); in lateral view post-petiole slightly dilated, 1.5 times larger than petiole (Fig. 23B, F); bicolored with head, mesosoma, and legs reddish yellow and gaster dark brown (Fig. 23B)	**Monomoriumcf.pharaonis**
8	Antennal scrobes very well marked, extending posteriorly past the eyes (Fig. 37D); frontal carina clearly differentiated to extending tenuously until or near to the occipital corners (Fig. 41C) (*Tetramorium*)	**9**
–	Antennal scrobes absent (Figs 15C, 28C); frontal carinae short and never extending posteriorly past the eyes (Figs 13C, 28D)	**13**
9	Propodeal spines long, strong, and acute (Figs 37C, 41D)	**10**
–	Propodeal spines short and not acute (Figs 38C, 40C)	**12**
10	Sculpture on the cephalic dorsum of the head strigose (Fig. 41E); body dark brown to black; legs, antennae, and mandibles pale brown (Fig. 41A)	***Tetramoriumlucayanum***
–	Sculpture on the cephalic dorsum of the head alveolate or areolate (Fig. 37E); yellowish and reddish ants (Figs 37B, 38B, 39B, 40B)	**11**
11	Anterior clypeal margin with a distinct median notch or impression; median portion of the clypeus with 3 longitudinal carinae (Fig. 37E); head, mesosoma, waist and gaster covered by numerous thick erect and suberect hairs (Fig. 37B, C, F); bicolored with gaster dark (Fig. 37B)	***Tetramoriumbicarinatum***
–	Anterior clypeal margin without a median notch or impression (Fig. 39C); median portion of the clypeus with a central carina weak or discontinuously marked (Fig. 39B); head, mesosoma, waist, and gaster densely covered by a fine and long white pilosity (Fig. 39D, E, F); entirely reddish (Fig. 39B)	***Tetramoriumlanuginosum***
12	Frontal carinae very well marked (Fig. 40D); antennal scrobes shallow, broad and conspicuous (Fig. 40E) mesosoma with < 10 erect hairs	***Tetramoriumsimillimum***
–	Frontal carinae not well marked, scrobes vestigial, feebly developed (Fig. 38D, E); mesosoma with > 10 erect hairs	***Tetramoriumcaldarium***
13	Head in full-face view and mesosoma in dorsal view strigose (Fig. 28C, E); mesosoma in lateral view clearly convex, without sutures impressed on the dorsum (Fig. 28B, E); eyes composed of 5 ommatidia (Fig. 28D) (*Rogeria*)	***Rogeriacurvipubens***
–	Head in full-face view and mesosoma in dorsal view with variable sculpturing, but never uniformly strigose; mesosoma with notopropodeal suture present and grooved in lateral view (Slightly reduced to absent in *Cardiocondylaminutior*); number of ommatidia variable	**14**
14	Monomorphic worker caste; dorsal view of the head and mesosoma densely foveolate with small appressed hairs (Figs 15C, D, 16D, F); promesonotum flat or slightly convex (Figs 15E, 16C); anterior margin of clypeus projected over the basal margin of the mandibles (Fig. 16E); in dorsal view, post-petiole spherical and notably dilated in comparison with petiole (Fig. 15F) *Cardiocondyla*)	**15**
–	Polymorphic worker caste; dorsal view of the head with the occipital corners smooth and shiny (major workers) (Fig. 27C); promesonotum convex (Fig. 25C); anterior margin of clypeus not projected over the basal margin of the mandibles (Fig. 25D); postpetiole never spherical (Fig. 26C) (*Pheidole*)	**16**
15	Metanotal groove not impressed on the dorsum of mesosoma (Fig. 16C, F); head, mesosoma, and gaster dark brown; propodeal spines short (Fig. 16C, B)	***Cardiocondylaminutior***
–	Metanotal groove impressed on the dorsum of mesosoma (Fig. 15D, E); mesosoma pale brown or orange, contrasting with darker gaster; propodeal spines longer and more acute than above (Fig. 15E)	***Cardiocondylaemeryi***
16	Major workers orange to reddish; total length ~ 2 mm (Figs 24B, 27B)	**17**
–	Major workers dark brown to brown; total length ~ 2.5 mm (Figs 25B, 26B)	**18**
17	Major workers: head in frontal view with antennal scrobe weakly developed and alveolate (Fig. 24D, E); mesosoma in lateral view alveolate (Fig. 24C, E)	***Pheidoleflavens***
–	Major workers: head in frontal view with antennal scrobe absent (Fig. 27A, C, B); mesosoma in lateral view, with the pronotum in major proportion smooth and shiny (Fig. 27B, D), but rugulose and alveolate between the mesonotum and propodeum (Fig. 27D)	***Pheidole* hh01**
18	Promesonotum in lateral view convex until it reaches the metanotal groove (Fig. 25C); post-petiole hexagonal in dorsal view exaggeratedly swollen relative to petiole (Fig. 25E); subpostpetiolar process slightly bulging (Fig. 25F)	***Pheidolemegacephala***
–	Promesonotum in lateral view forming two convexities, truncated before reaching the metanotal groove (Fig. 26D); postpetiole not swollen compared to petiole (Fig. 26E); subpostpetiolar process absent or reduced (Fig. 26F)	***Pheidolewilliamsi***
19	Antenna with 10 or 11 segments (Figs 29C, 43C)	**20**
–	Antenna with < 6 segments (Fig. 33F), (*Strumigenys*)	**24**
20	Antenna with 10 segments; funiculus with 2-segmented club (Fig. 29C); antennal scrobes absent (Fig. 30C); propodeum without spines (Fig. 31C) (*Solenopsis*)	**21**
–	Antenna with 11 segments (Figs 18C, 43E); funiculus with a diffuse 3-segmented club (Figs 18C, 43C) antennal scrobes present (Figs 19E, 43E); spines on propodeum present or not (Figs 20C, 43D)	**27**
21	Large (Fig. 29B), second and usually third segments of funiculus at least 1½ times as long as broad (Fig. 29A, C); petiole with thin flange ventrally; dark brown to black (Fig. 29B)	***Solenopsisgeminata***
–	Smaller (Figs 30B, 31B, 32B), second and third segments of funiculus at most only slightly longer than broad, usually broader than long (Fig. 31D); petiole lacking flange ventrally, reddish to orange and dark brown (Figs 30B, 31B, 32B)	**22**
22	Postpetiole greatly dilated, wider than petiole (seen from above), globose; eye with 15–25 ommatidia (Fig. 30D, E)	***Solenopsisglobularia***
–	Postpetiole not dilated nor globose (Fig. 32C); eye with 3–5 ommatidia (Fig. 31D)	**23**
23	In full face view, occipital margin of the head slightly concave (Fig. 31E); anterior clypeal margin with the median portion concave and oriented onward; frontal lobes longitudinally striated (Fig. 31E)	***Solenopsisgnoma***
–	In full face view, occipital margin of the head relatively straight to convex rather than concave (Fig. 32E); anterior clypeal margin with the median portion erect and not oriented onward; frontal lobes smooth and shiny, not striated (Fig. 32E)	**Solenopsiscf.basalis (hh06)**
24	Mandibles long and straight (Figs 33C, 35C)	**25**
–	Mandibles short and curved downwards in profile, otherwise triangular (Figs 34D, 36C)	**26**
25	Mandibles with a small preapical tooth, without denticles on inner border (Fig. 35C); head and mesosoma with appressed spatulate hairs (Figs 34E, 35D, E, F)	***Strumigenyslouisianae***
–	Mandibles armed with small denticles on inner border (Fig. 33C); head and mesosoma without appressed (spatulate) circular hairs (Fig. 33D, E)	***Strumigenyseggersi***
26	Triangular mandibles armed with denticles (Fig. 36A); antenna with 6 segments, head and mesosoma with few appressed hairs (Fig. 36D); petiole and post-petiole with spongiform tissue (Fig. 36E)	***Strumigenysmembranifera***
–	Mandibles in appearance curved and short, armed with an apical fork (Fig. 33D); antenna with four segments (Fig. 33C); head and mesosoma with appressed spatulate hairs (Fig. 33E); petiole and post-petiole without spongiform tissue	***Strumigenysemmae***
27	Head in frontal view with the antennal insertions hidden under the frontal lobes, which are exceptionally broad or expanded (Figs 18C, 20A, D); promesonotum tuberculated and propodeum unarmed by spines (Figs 18D; 19C, 20C); first segment of the gaster covered with appressed hairs, dull and opaque (Figs 19E, 20E) (*Cyphomyrmex*)	**28**
–	Head in full face view with the antennal insertions partly visible (Fig. 43A); frontal lobes not distended; promesonotum not tuberculated (Fig. 43D); propodeum armed with a pair of acute spines (Fig. 43D); first segment of the gaster smooth and shiny with few erect setae (Fig. 43F) (*Wasmannia*)	***Wasmanniaauropunctata***
28	Pair of tubercles absent in the anterior median region of the pronotum (Fig. 18E); dark brown (Fig. 18B)	***Cyphomyrmexnesiotus***
–	Pair of tubercles present in the anterior median region of the pronotum (Figs 19D, 20E)	**29**
29	In dorsal view, propodeal declivity with a pair of tubercles located at the level of spiracles (Fig. 20E, F); head and body black (Fig. 20B)	***Cyphomyrmex* sp. hh04**
–	In dorsal view, propodeal declivity without a pair of tubercles situated at level of spiracles (Fig. 19C, D); head and gaster brown, mesosoma and legs pale brown (Fig. 19B)	***Cyphomyrmexrimosus***

### ﻿Genus *Adelomyrmex* Emery, 1897

***Adelomyrmexlonginoi* Fernández, 2003**

Fig. 14

**Remarks.** Misidentification in Herrera and Longino (2008). Cited in Longino (2012) and Herrera (2015, 2019) [ICCDRS].

**Figure 14.**
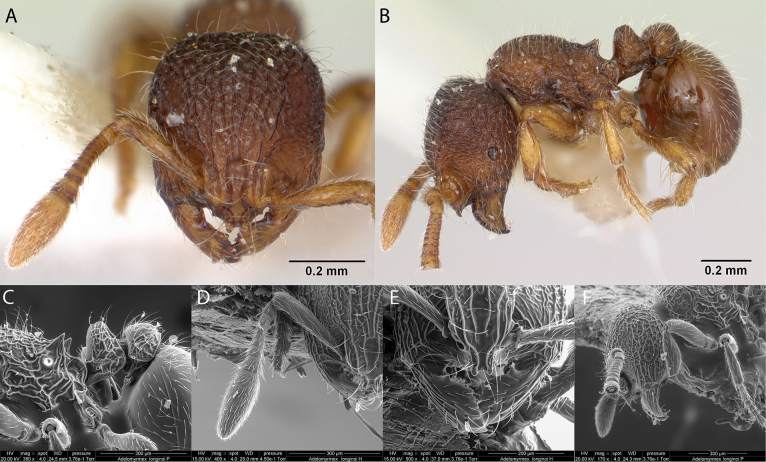
*Adelomyrmexlonginoi* worker micrographs in **A** head in full-face view **B** view in profile, and SEM images of **C** propodeum, petiole and postpetiole in profile **D** antennae in front view **E** mandibles in front view **F** head in profile.

**Taxonomic history.**Bolton (1995, 2014), Bolton et al. (2006).

**Distribution.** Central America.

**Galápagos distribution.** Introduced: Isabela Island (Herrera et al. 2014).

### ﻿Genus *Cardiocondyla* Emery, 1869

***Cardiocondylaemeryi* Forel, 1881**

Fig. 15

**Remarks.** Cited in Lubin (1984), Lubin (1985), Pezzatti et al. (1998), Roque-Albelo et al. (2000) [ICCDRS], von Aesch and Cherix (2005), von Aesch (2006) [ICCDRS], Causton et al. (2006), McMullen (2007), Dekoninck et al. (2014) [ICCDRS, RBINS], Wauters et al. (2016) [RBINS], Herrera (2015, 2019) and Herrera et al. (2020) [ICCDRS; RBINS]. Probably *C.minutior* or *C.emeryi* in Peck (1994a) and Peck (1994b).

**Figure 15.**
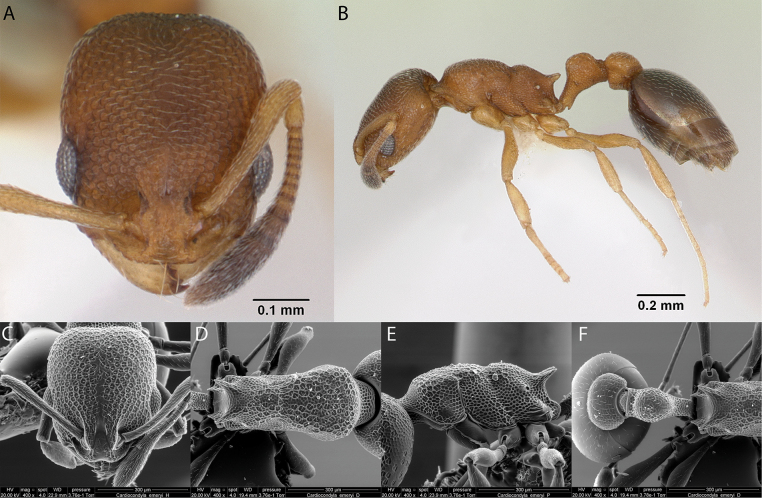
*Cardiocondylaemeryi* worker micrographs in **A** head in full-face view **B** view in profile, and SEM images of **C** head in full-face view **D** mesosoma in dorsal view **E** mesosoma in profile **F** propodeum, petiole and postpetiole in dorsal view.

**Taxonomic history.**Bolton (1995, 2014), Bolton et al. (2006).

**Distribution.** Afrotropical, Australasia, Indomalaya, Malagasy, Nearctic, Neotropical, Oceania, Palearctic.

**Galápagos distribution.** Introduced: Albany, Bainbridge #1, Bainbridge #3, Bainbridge #4, Bainbridge #5, Bainbridge #6, Bainbridge #8, Bar, Cousin, Darwin, Eden, Fernandina, Floreana, Gardner (next to Floreana), Genovesa, Gran Felipe, Isabela (CA, SN, VA, VD, VE, VW), Mariela Grande, Mao, Marchena, Pinta, Pinzón, Plaza Sur, Rábida, Santiago, San Cristóbal, Santa Cruz, Seymour Norte, Santa Fé, Wolf (Herrera et al. 2020).

***Cardiocondylaminutior* Forel, 1899**

Fig. 16

**Remarks.** Cited as *Cardiocondylanuda* in Lubin (1984), Lubin (1985), [ICCDRS], Roque-Albelo et al. (2000) [ICCDRS], Pezzatti et al. (1998) [ICCDRS], von Aesch and Cherix (2005), von Aesch (2006) [ICCDRS]. Cited as *C.nuda* in Causton et al. (2006). Probably *C.minutior* in McMullen (1993). *Cardiocondylaminutior* or *C.emeryi* in Peck (1994a, 1994b). *Cardiocondylaminutior* in Wauters et al. (2016), Herrera (2015, 2019) and Herrera et al. (2020) [ICCDRS].

**Figure 16.**
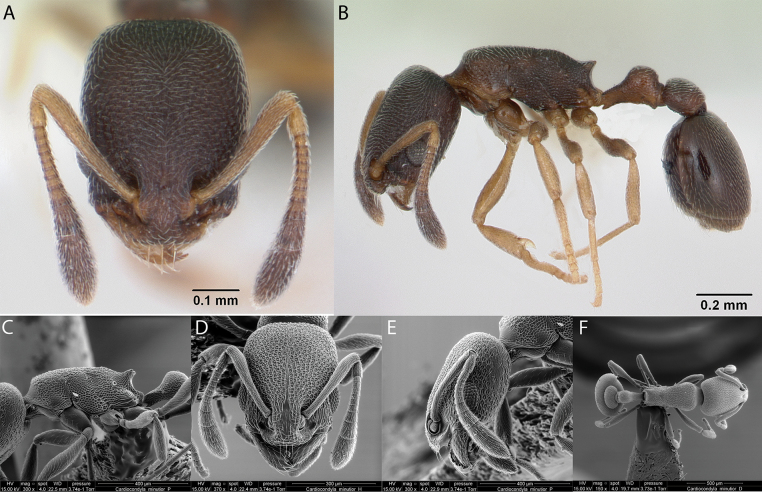
*Cardiocondylaminutior* worker micrographs in **A** head in full-face view **B** view in profile, and SEM images of **C** mesosoma in profile **D** head in full-face view **E** head in profile **F** mesosoma in dorsal view.

**Taxonomic history.**Bolton (1995, 2014), Bolton et al. (2006).

**Distribution.** Afrotropical, Australasia, Indomalaya, Malagasy, Nearctic, Neotropical, Oceania.

**Galápagos distribution.** Introduced: Bainbridge #1, Cousin, Daphne Mayor, Darwin, Fernandina, Floreana, Gardner (next to Floreana), Isabela (CA, SN, VA, VD, VE), Mariela Grande, Marchena, Pinta, Santiago, San Cristóbal, Santa Cruz, Santa Fé, Wolf (Herrera et al. 2020).

### ﻿Genus *Crematogaster* Lund, 1831

***Crematogastercrinosa* Mayr, 1862**

**Remarks.** Cited as Crematogaster (Orthocrema) brevispionsa chatamensis in Wheeler (1933), Kempf (1972), Linsley and Usinger (1966). *Crematogasterchatamensis* in Lubin (1984). *Crematogastercrinosa* in Longino (2003) [CAS], Herrera et al. (2014), Herrera (2015, 2019) and Herrera et al. (2020).

**Taxonomic history.**Bolton (1995, 2014), Bolton et al. (2006).

**Distribution.** Neotropical.

**Galápagos distribution.** Uncertain: San Cristóbal (Wheeler 1933).

### ﻿Genus *Crematogaster* Lund, 1831

***Crematogaster* JTL-022**

Fig. 17

**Remarks.** First published record Herrera et al. (2014), cited also in Traveset et al. (2013), Herrera (2015, 2019) and Herrera et al. (2020) [ICCDRS, JTLC].

**Figure 17.**
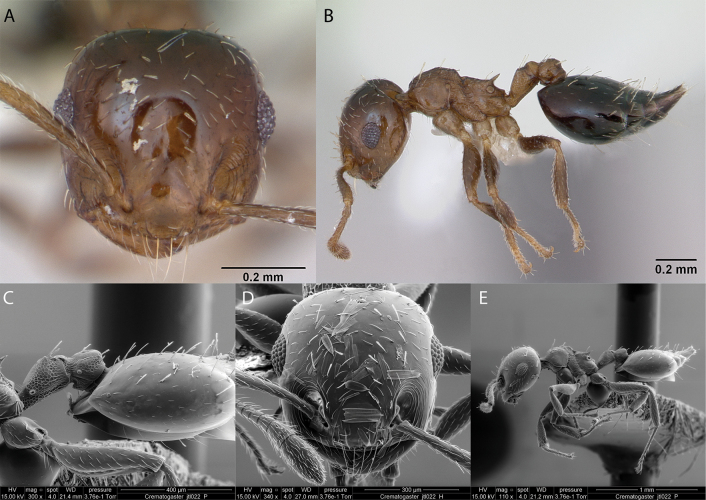
*Crematogaster* JTL-022 worker micrographs in **A** head in full-face view **B** view in profile, and SEM images of **C** petiole and postpetiole in profile **D** head in full-face view **E** view in profile.

**Distribution.** Introduced: San Cristóbal Island (Herrera et al. 2014, 2020).

### ﻿Genus *Cyphomyrmex* Mayr, 1862

***Cyphomyrmexnesiotus* Snelling & Longino, 1992**

Fig. 18

**Remarks.** Cited in Snelling and Longino (1992), Herrera and Longino (2008), Herrera (2015, 2019) and Herrera et al. (2020) [ICCDRS, JTLC].

**Figure 18.**
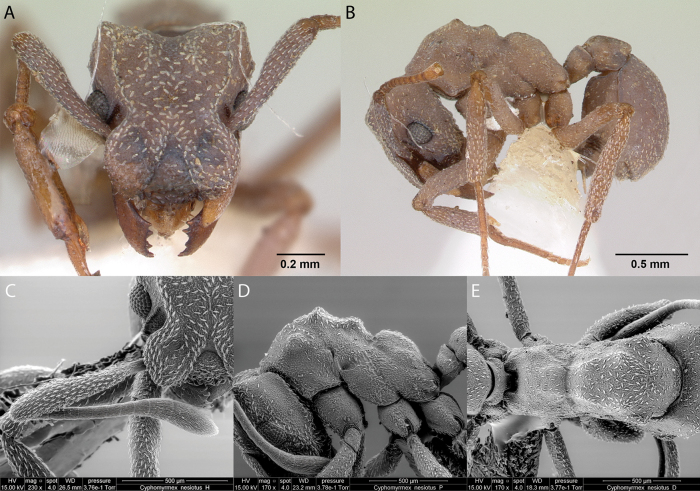
*Cyphomyrmexnesiotus* worker micrographs in **A** head in full-face view **B** view in profile, and SEM images of **C** antennae in front view **D** mesosoma in profile **E** mesosoma in dorsal view.

**Taxonomic history.**Bolton (1995, 2014), Bolton et al. (2006).

**Distribution.** Probably endemic: Isabela (Snelling and Longino 1992).

***Cyphomyrmexrimosus* (Spinola, 1851)**

Fig. 19

**Remarks.** First published record Herrera and Longino (2008) [ICCDRS]. Cited also in Dekoninck et al. (2014), Wauters et al. (2016), Herrera (2015, 2019) and Herrera et al. (2020) [ICCDRS]. Probably *C.rimosus* in Lubin (1984) and Brandão and Paiva (1994).

**Figure 19.**
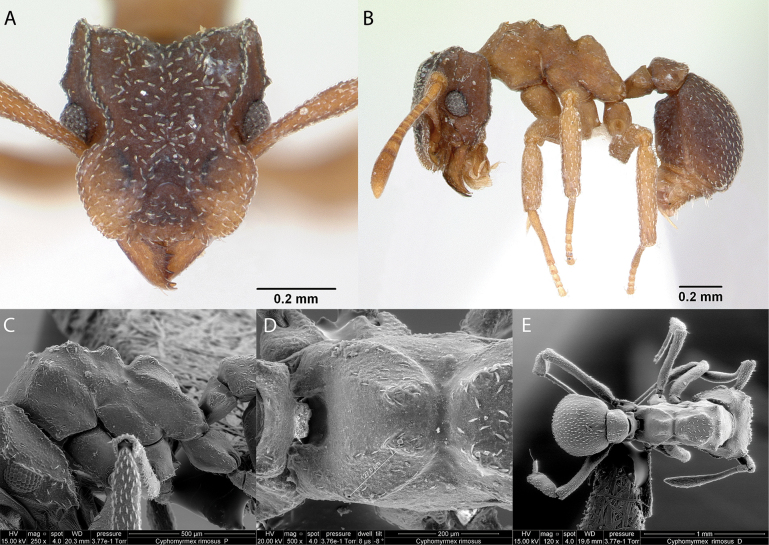
*Cyphomyrmexrimosus* worker micrographs in **A** head in full-face view **B** view in profile, and SEM images of **C** head and mesosoma in profile **D** propodeum in dorsal view **E** dorsal view.

**Taxonomic history.**Bolton (1995, 2014), Bolton et al. (2006).

**Distribution.** Nearctic and Neotropical.

**Galápagos distribution.** Introduced: Gardner (next to Floreana), Isabela (SN), San Cristóbal, Santa Cruz (Herrera et al. 2020).

***Cyphomyrmex* sp. hh04**

Fig. 20

**Remarks.** First published record as dark form of *C.rimosus* in Herrera and Longino (2008). Cited as *Cyphomyrmex* sp. hh04 in Herrera (2015, 2019) and Herrera et al. (2020) [ICCDRS, RBINS].

**Figure 20.**
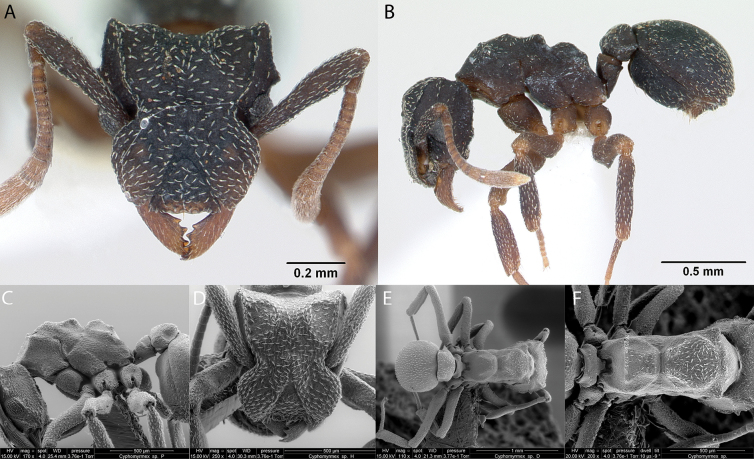
*Cyphomyrmex* sp. hh004 worker micrographs in **A** head in full-face view **B** view in profile, and SEM images of **C** mesosoma in profile **D** head in full-face view **E** dorsal view **F** mesosoma in dorsal view.

**Distribution.** Origin uncertain: Isabela (SN) Pinzón, Santa Cruz (Herrera et al. 2020).

**New record.** Santiago Island.

### ﻿Genus *Monomorium* Mayr, 1855

***Monomoriumfloricola* (Jerdon, 1851)**

Fig. 21

**Remarks.** Originally cited as *Attafloricola* in (Jerdon, 1851). Cited as *Monomoriumfloreanun* in Stitz (1932). *Monomoriumfloricola* in Linsley and Usinger (1966). *Monomoriumfloreanun* in Kempf (1972). *Monomoriumfloricola* in Kempf (1972), Clark et al. (1982), Lubin (1984) [ICCDRS], McMullen (1993), Meier (1994) [ICCDRS], Abedrabbo (1994) [ICCDRS], de la Vega (1994), Peck (1994a), Peck et al. (1998), Pezzatti et al. (1998) [ICCDRS], Roque-Albelo et al. (2000) [ICCDRS], von Aesch and Cherix (2005), Boada (2005) [ICCDRS], von Aesch (2006) [ICCDRS], Causton et al. (2006), Herrera and Causton (2010) [ICCDRS], McMullen (2012), Chamorro et al. (2012) [ICCDRS], Dekoninck et al. (2014) [ICCDRS], Wauters et al. (2016) [RBINS], Herrera (2015, 2019) and Herrera et al. (2020) [ICCDRS].

**Figure 21.**
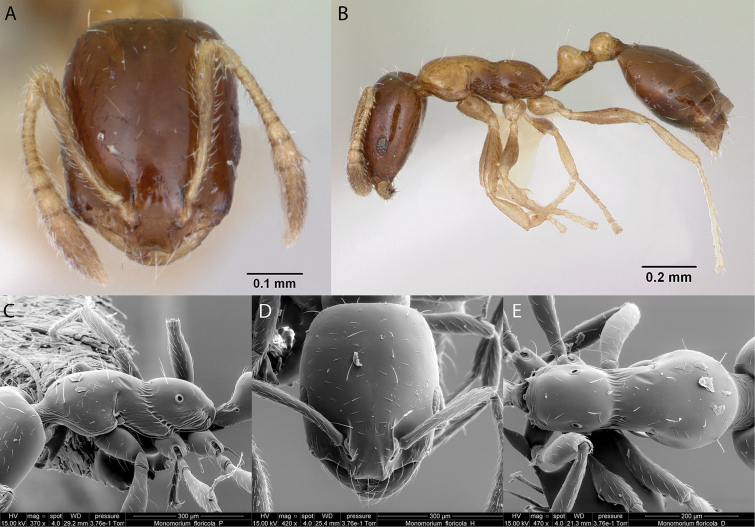
*Monomoriumfloricola* worker micrographs in **A** head in full-face view **B** view in profile, and SEM images of **C** mesosoma in profile **D** head in full-face view **E** mesosoma in dorsal view.

**Taxonomic history.**Bolton (1995, 2014), Bolton et al. (2006).

**Distribution.** Afrotropical, Australasia, Indomalaya, Malagasy, Nearctic, Neotropical, Oceania, Palearctic.

**Galápagos distribution.** Introduced: Bainbridge #5, Baltra, Bartolomé, Bayas, Bowditch South, Champion, Cousin, Daphne Mayor, Española, Fernandina, Floreana, Gardner (next to Floreana), Genovesa, Isabela (CA, SN, VA, VD), Mariela Grande, Mariela Mediana, Marchena, Pinta, Plaza Norte, Plaza Sur, Rábida, Santiago, San Cristóbal, Santa Cruz, Seymour Norte, Santa Fé (Herrera et al. 2020).

**New record.** Sombrero Chino.

***Monomoriumpharaonis* (Linnaeus, 1758)**

Fig. 22

**Remarks.** Originally cited as *Formicapharaonis* in (Linnaeus, 1758). Galápagos first published record in Wheeler (1919). Cited also in Linsley and Usinger (1966), Kempf (1972), Lubin (1984) [ICCDRS], Brandão and Paiva (1994), Peck et al. (1998), Causton et al. (2006), Herrera (2015, 2019), and Herrera et al. (2020) [ICCDRS].

**Figure 22.**
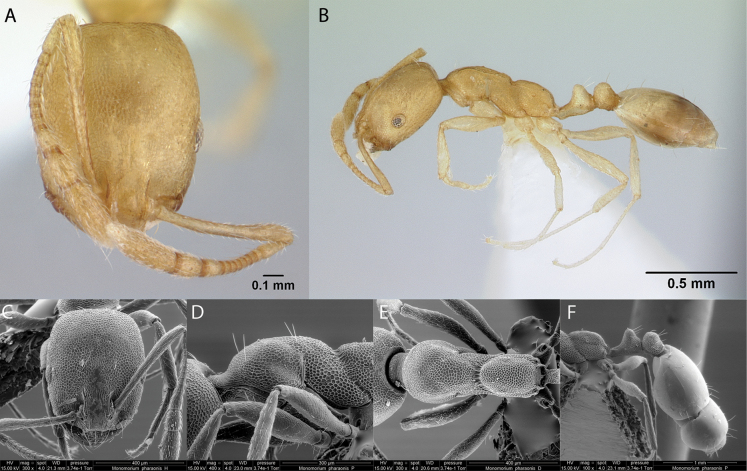
*Monomoriumpharaonis* worker micrographs in **A** head in full-face view **B** view in profile, and SEM images of **C** head in full-face view **D** mesosoma in profile **E** mesosoma in dorsal view **F** petiole and postpetiole in profile.

**Taxonomic history.**Bolton (1995, 2014), Bolton et al. (2006).

**Distribution.** Afrotropical, Australasia, Indomalaya, Malagasy, Nearctic, Neotropical, Oceania, Palearctic.

**Galápagos distribution.** Introduced: Baltra, Isabela (SN), Pinta, Santa Cruz (Herrera et al. 2020).

**Monomoriumcf.pharaonis**

Fig. 23

**Remarks.** First record in Herrera and Causton (2010) [ICCDRS]. Cited also in Dekoninck et al. (2014) [ICCDRS], Wauters et al. (2016) [ICCDRS], Herrera (2015, 2019) and Herrera et al. (2020) [ICCDRS].

**Figure 23.**
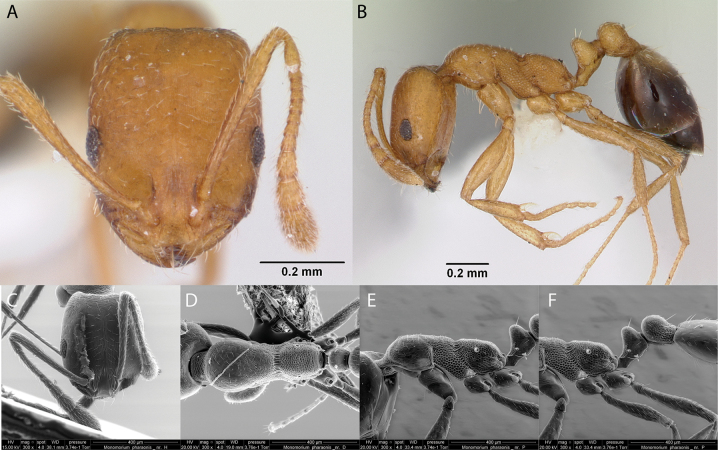
Monomorium sp. nr. pharaonis worker micrographs in **A** head in full-face view **B** view in profile, and SEM images of **C** head in full-face view **D** mesosoma in dorsal view **E** mesosoma in profile **F** petiole and postpetiole in profile.

**Distribution.** Undetermined origin: Baltra, Fernandina, Floreana, Isabela (SN), Marchena, Pinta, San Cristóbal, Santa Cruz, Santa Fé (Herrera et al. 2020).

### ﻿Genus *Pheidole* Westwood, 1839

***Pheidoleflavens* Roger, 1863**

Fig. 24

**Remarks.** Cited in Wheeler (1919), Clark et al. (1982), Herrera et al. (2014) [ICCDRS], Herrera (2015, 2019) and Herrera et al. (2020) [ICCDRS].

**Figure 24.**
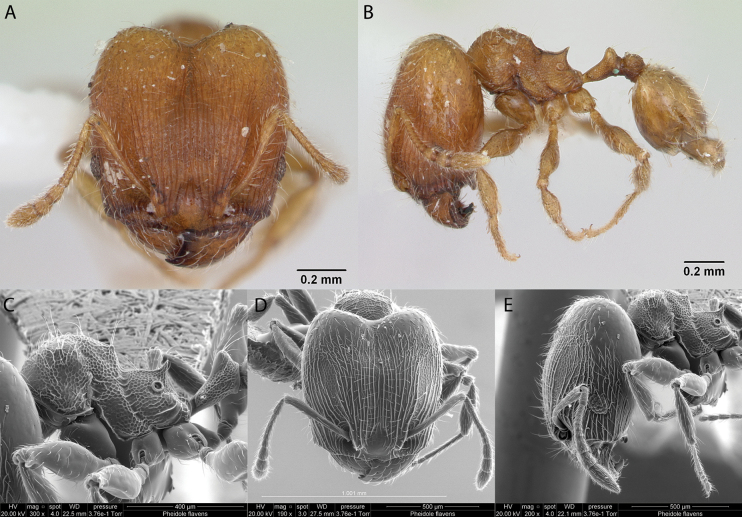
*Pheidoleflavens* worker micrographs in **A** head in full-face view **B** view in profile, and SEM images of **C** mesosoma in profile **D** head in full-face view **E** head in profile.

**Taxonomic history.**Bolton (1995, 2014), Bolton et al. (2006).

**Distribution.** Neotropical.

**Galápagos distribution.** Introduced: Isabela (CA, SN, VA, VD, VW), San Cristóbal, Santa Cruz (Herrera et al. 2020).

***Pheidolemegacephala* (Fabricius, 1793)**

Fig. 25

**Remarks.** Originally cited as *Formicamegacephala* (Fabricius, 1793). Cited in Herrera et al. (2013) [ICCDRS], Wauters et al. (2016) [RBINS], Herrera (2015, 2019) and Herrera et al. (2020).

**Figure 25.**
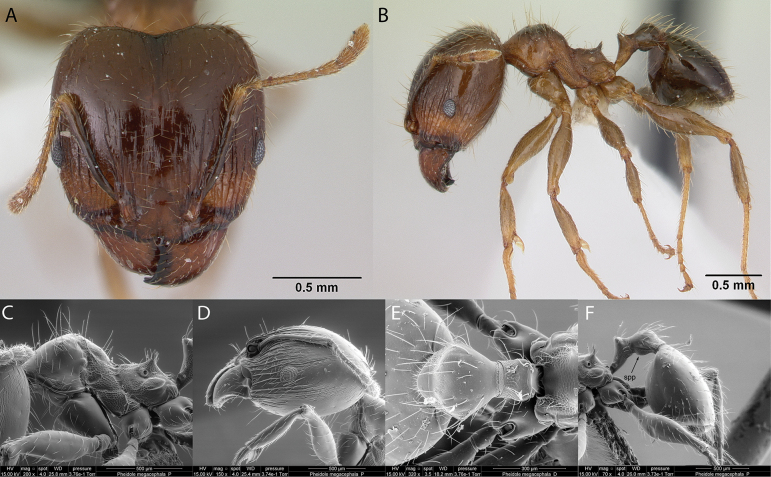
*Pheidolemegacephala* worker micrographs in **A** head in full-face view **B** view in profile, and SEM images of **C** mesosoma in profile **D** head in profile **E** petiole and postpetiole in dorsal view **F** petiole and postpetiole in profile (spp = subpetiolar process).

**Taxonomic history.**Bolton (1995, 2014), Bolton et al. (2006).

**Distribution.** Afrotropical, Australasia, Indomalaya, Malagasy, Nearctic, Neotropical, Oceania, Palearctic.

**Galápagos distribution.** Introduced: Isabela (SN), San Cristóbal, Santa Cruz (Herrera et al. 2013).

***Pheidolewilliamsi* Wheeler, 1919**

Fig. 26

**Remarks.** Cited as *Pheidolewilliamsi* in (Wheeler 1919). Pheidolewilliamsivar.seymourensis in Wheeler (1924), Linsley and Usinger (1966). *Pheidolewilliamsiwilliamsi* in Linsley and Usinger (1966). *Pheidolewilliamsi* in Clark et al. (1982), Lubin (1984, 1985), Espadaler (1997), Wilson (2003), Herrera et al. (2014) [ICCDRS], Herrera (2015, 2019), and Herrera et al. (2020) [ICCDRS].

**Figure 26.**
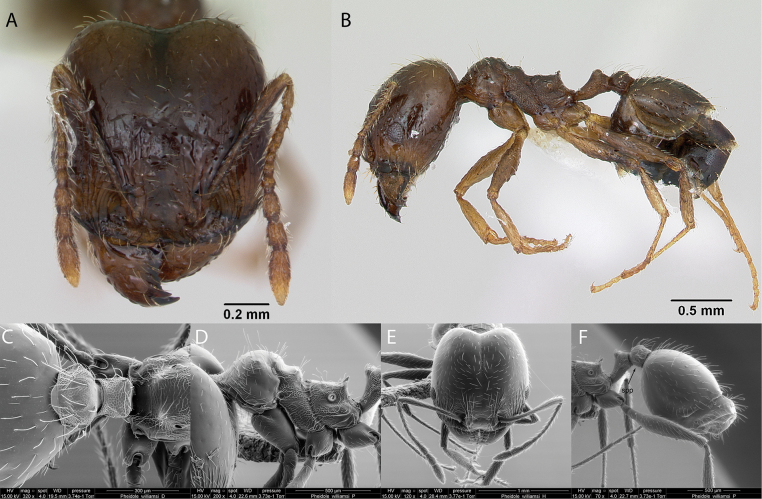
*Pheidolewilliamsi* worker micrographs in **A** head in full-face view **B** view in profile, and SEM images of **C** petiole and postpetiole in profile **D** mesosoma in profile **E** head in profile **F** petiole and postpetiole in profile (spp = subpetiolar process).

**Taxonomic history.**Kempf (1972), Bolton (1995, 2014), Bolton et al. (2006).

**Distribution.** Possibly endemic: Albany, Bainbridge #1, Bainbridge #2, Bainbridge #3, Bainbridge #4, Bainbridge #5, Bainbridge #6, Baltra, Bowditch South, Daphne Mayor, Fernandina, Floreana, Gardner (next to Floreana), Isabela (SN, VA, VD, VW), Mariela Grande, Mariela Mediana, Pinta, Plaza Sur, Rábida, Santiago, San Cristóbal, Santa Cruz, Seymour Norte, Santa Fé, Tortuga (Herrera et al. 2020).

**New records.** Bartolomé and Beagle.

***Pheidole* sp. hh01**

Fig. 27

**Remarks.** In Herrera et al. (2014), Herrera (2015, 2019), and Herrera et al. (2020) [ICCDRS].

**Figure 27.**
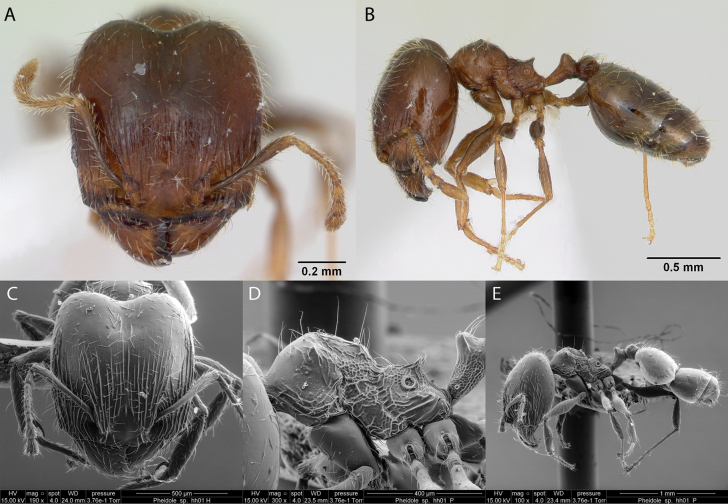
*Pheidole* sp. hh01 worker micrographs in **A** head in full-face view **B** view in profile, and SEM images of **C** head in full-face view **D** mesosoma in profile **E** view in profile.

**Distribution.** Origin uncertain: Bowditch South, Eden, Floreana, Isabela (CA, SN, VA, VD, VE, VW), Logie, Pinzón, Santiago, San Cristóbal, Santa Cruz (Herrera et al. 2020).

### ﻿Genus *Rogeria* Emery, 1894

***Rogeriacurvipubens* Emery, 1894**

Fig. 28

**Remarks.** Galápagos first published record (Herrera and Longino 2008), cited also in Dekoninck et al. (2014), Wauters et al. (2016), Herrera (2015, 2019), and Herrera et al. (2020) [ICCDRS].

**Figure 28.**
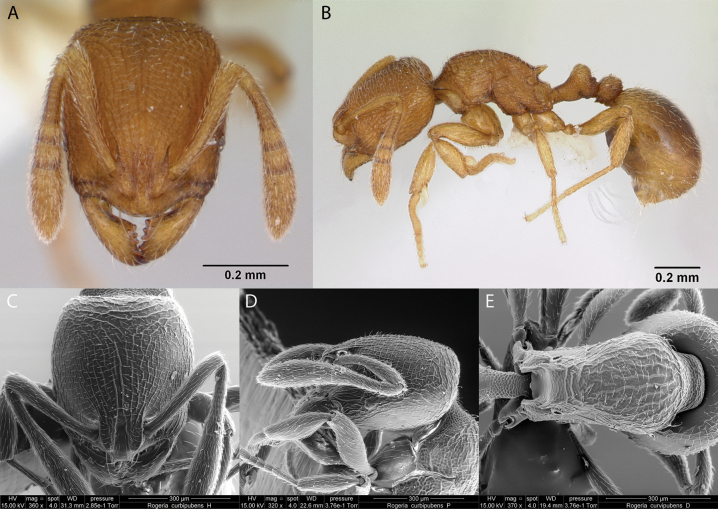
*Rogeriacurvipubens* worker micrographs in **A** head in full-face view **B** view in profile, and SEM images of **C** head in full-face view **D** head in profile **E** mesosoma in dorsal view.

**Taxonomic history.**Bolton (1995, 2014), Bolton et al. (2006).

**Distribution.** Neotropical.

**Galápagos distribution.** Introduced: Isabela (SN), San Cristóbal, Santa Cruz (Herrera et al. 2020).

### ﻿Genus *Solenopsis* Westwood, 1840

***Solenopsisgeminata* (Fabricius, 1804)**

Fig. 29

**Remarks.** Originally cited as *Attageminata* in (Fabricius, 1804). Cited as *Solenopsisgeminata* in Emery (1893). *Solenopsisgeminatagalapageia* in Wheeler (1919), Linsley and Usinger (1966), and Kempf (1972). *Solenopsisgeminata* in Lubin (1984), Williams (1987), Trager (1991), Williams and Whelan (1991), Brandão and Paiva (1994), Meier (1994), de la Vega (1994), Peck et al. (1998), Pezzatti et al. (1998), von Aesch and Cherix (2005) [ICCDRS], Boada (2005) [ICCDRS], von Aesch (2006) [ICCDRS], Causton et al. (2006), Pacheco et al. (2007), Herrera and Causton (2010) [ICCDRS], Herrera and Longino (2008), Herrera and Causton (2010) [ICCDRS], Herrera et al. (2013), Dekoninck et al. (2014) [ICCDRS, RBINS], Wauters et al. (2014) [RBINS], Wauters et al. (2016), [RBINS] Herrera (2015, 2019), and Herrera et al. (2020) [ICCDRS].

**Figure 29.**
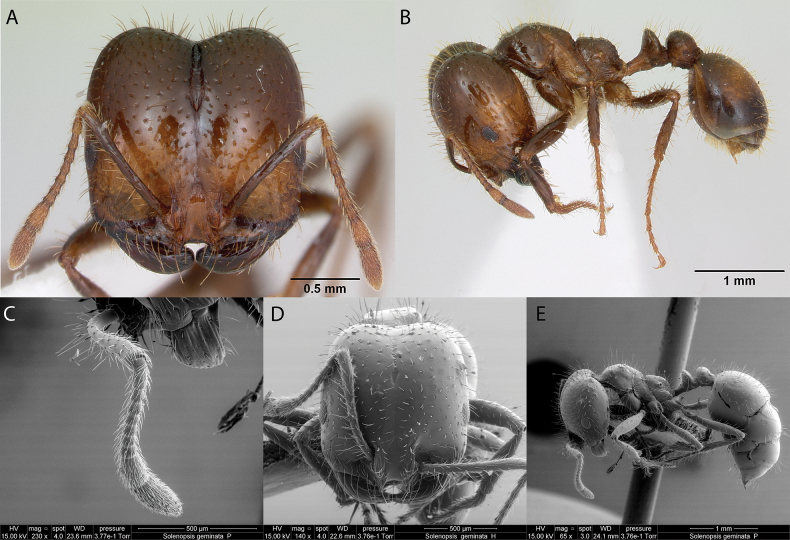
*Solenopsisgeminata* worker micrographs in **A** head in full-face view **B** view in profile, and SEM images of **C** antennae funiculi in profile **D** head in full-face view **E** view in profile.

**Taxonomic history.**Trager (1991), Bolton (1995, 2014), Bolton et al. (2006).

**Distribution.** Afrotropical, Australasia, Indomalaya, Malagasy, Nearctic, Neotropical, Oceania, Palearctic.

**Galápagos distribution.** Introduced: Albany, Bainbridge #1, Baltra, Bayas, Champion, Cuevas, Eden, Enderby, Fernandina, Floreana, Gardner (next to Floreana), Isabela (CA, SN, VA), Mariela Grande, Mao, Mariela Mediana, Plaza Sur, Santa Fé, Santiago, San Cristóbal, Santa Cruz, Seymour Norte (Herrera et al. 2020).

***Solenopsisglobularia* (Smith, 1858)**

Fig. 30

**Remarks.** Originally cited as *Myrmicaglobulariapacifica* in (Smith, 1858). Cited as *Solenopsisglobulariapacifica* in Wheeler (1919, 1924). Solenopsisglobulariapacificavar.rubida in Wheeler (1919, 1924), *Solenopsisglobulariapacifica* in Linsley and Usinger (1966). *Solenopsisglobulariarubida* in Linsley and Usinger (1966). *Solenopsisglobulariapacifica* and Solenopsisglobulariapacificavar.rubida in Kempf (1972). *Solenopsisglobularia* in Clark et al. (1982). *Solenopsispacifica* in Lubin (1984). *Solenopsisglobularia* in Lubin (1985) [ICCDRS], Meier (1994), Abedrabbo (1994) [ICCDRS], Peck et al. (1998), (Pezzatti et al. (1998), Roque-Albelo et al. (2000) [ICCDRS], von Aesch and Cherix (2005), von Aesch (2006), Causton et al. (2006), Pacheco et al. (2007), Herrera and Causton (2010) [ICCDRS], McMullen (2012), Pacheco and Mackay (2013), Herrera (2015, 2019), and Herrera et al. (2020) [ICCDRS].

**Figure 30.**
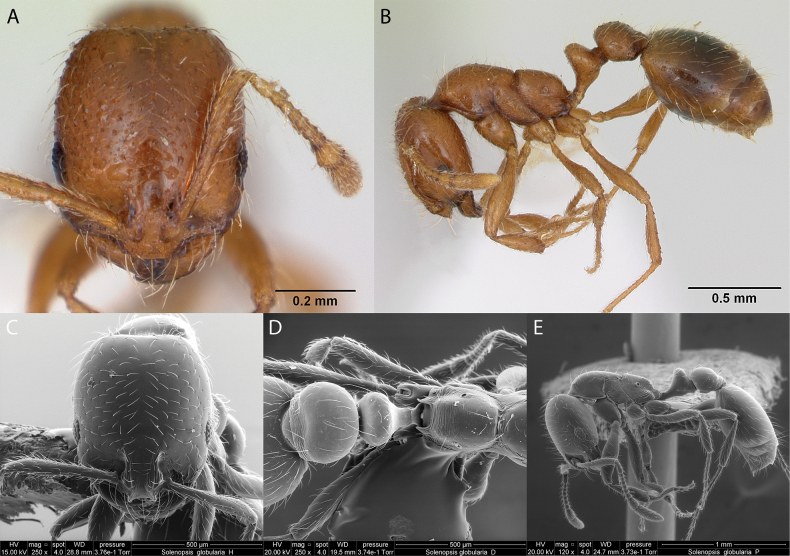
*Solenopsisglobularia* worker micrographs in **A** head in full-face view **B** view in profile, and SEM images of **C** head in full-face view **D** petiole and postpetiole in dorsal view **E** head and mesosoma in profile.

**Taxonomic history.**Bolton (1995, 2014), Bolton et al. (2006), Pacheco and Mackay (2013).

**Distribution.** Afrotropical, Nearctic, Neotropical.

**Galápagos distribution.** Introduced: Albany, Bainbridge #1, Bainbridge #3, Bainbridge #5, Bainbridge #7, Bainbridge #8, Baltra, Bowditch South, Champion, Daphne Mayor, Darwin, Eden, Enderby, Española, Fernandina, Floreana, Gardner (next to Española), Gardner (next to Floreana), Genovesa, Isabela (CA, SN, VA, VD, VE, VW), Mariela Grande, Mao, Mariela Pequeña, Marchena, Pinta, Pinzón, Plaza Sur, Rábida, Santiago, San Cristóbal, Santa Cruz, Seymour Norte, Santa Fé, Tortuga (Herrera et al. 2020).

**New record.** Sombrero Chino.

***Solenopsisgnoma* Pacheco, Herrera & Mackay, 2007**

Fig. 31

**Remarks.** Cited also in Dekoninck et al. (2014), Wauters et al. (2016), Herrera (2015, 2019) and Herrera et al. (2020) [ICCDRS].

**Figure 31.**
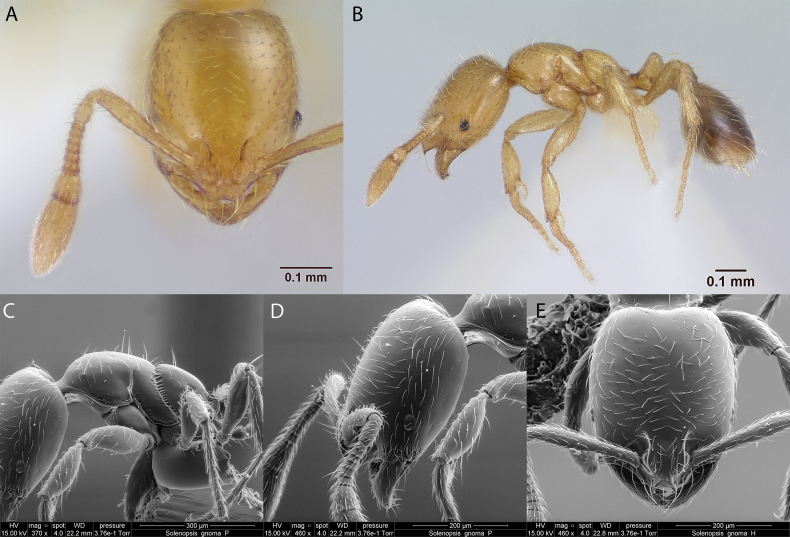
*Solenopsisgnoma* worker micrographs in **A** head in full-face view **B** view in profile, and SEM images of **C** mesosoma in profile **D** head in profile **E** head in full-face view.

**Taxonomic history.**Pacheco and Mackay (2013), Bolton (2014).

**Distribution.** Probably endemic: Albany, Bowditch South, Española, Floreana, Isabela (SN, VA, CA), Marchena, San Cristóbal, Santa Cruz (Pacheco et al. 2007; Herrera et al. 2020).

**New record.** Santiago.

***Solenopsissaevissima* (Smith**, **1855)**

**Remarks.** Originally cited as *Myrmicasaevissima* (Smith, 1855). Doubtful record for Galápagos (Herrera et al. 2020). Cited in Wheeler (1919, 1924), Linsley and Usinger (1966) and Brandão and Paiva (1994), probably misidentification in Peck et al. (1998). Cited also from literature in Causton et al. (2006).

**Taxonomic history.**Bolton (1995, 2014), Bolton et al. (2006).

**Distribution.** Nearctic, Neotropical.

**Galápagos distribution.** Uncertain: Santa Cruz Island (Wheeler 1919; Peck et al. 1998).

**Solenopsiscf.basalis (hh06)**

Fig. 32

**Remarks.** First record in Herrera et al. (2014), Herrera (2015, 2019) and Herrera et al. (2020) [ICCDRS].

**Figure 32.**
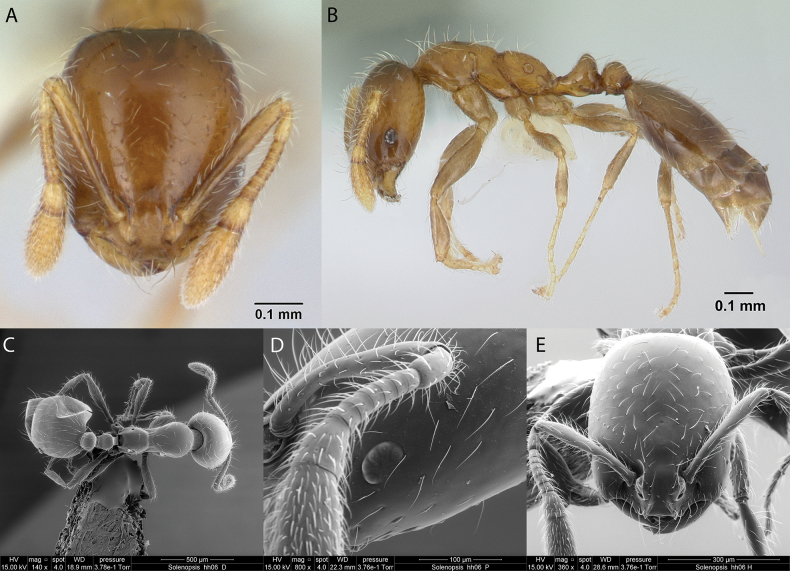
*Solenopsis* sp. basalis (hh06) worker micrographs in **A** head in full-face view **B** view in profile, and SEM images of **C** mesosoma in profile **D** head in profile **E** head in full-face view.

**Distribution.** Origin uncertain: Bainbridge #5, Santa Cruz, Santiago (Herrera et al. 2014).

### ﻿Genus *Strumigenys* Smith, 1860

***Strumigenyseggersi* Emery, 1890**

Fig. 33

**Remarks.** Galápagos first published record in Herrera et al. (2014). Cited also in Herrera (2015, 2019) and Herrera et al. (2020) [ICCDRS].

**Figure 33.**
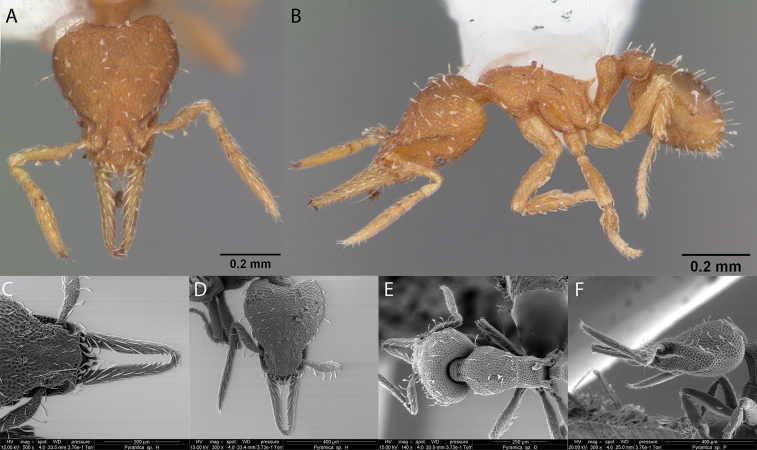
*Strumigenyseggersi* worker micrographs in **A** head in full-face view **B** view in profile, and SEM images of **C** close-up on mandibles **D** head in full-face view **E** mesosoma in dorsal view **F** head in profile.

**Taxonomic history.**Bolton (1995, 2014), Bolton et al. (2006).

**Distribution.** Indomalaya, Nearctic, Neotropical.

**Galápagos distribution.** Introduced: Santa Cruz (Herrera et al. 2014).

***Strumigenysemmae* (Emery, 1890)**

Fig. 34

**Remarks.** Originally cited as *Epitritusemmae* in (Emery, 1890). Cited as *Quadristrumaemmae* in Pezzatti et al. (1998) and Causton et al. (2006). Also, in Herrera et al. (2014). Wauters et al. (2016), Herrera et al. 2020) [ICCDRS].

**Figure 34.**
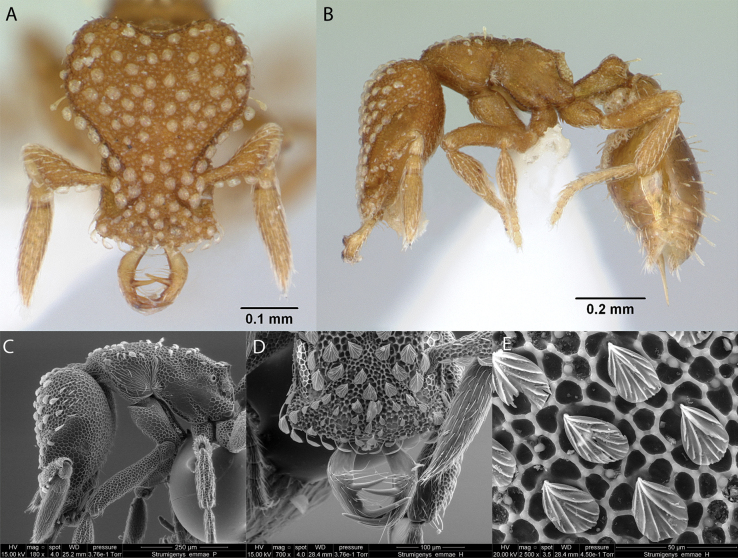
*Strumigenysemmae* worker micrographs in **A** head in full-face view **B** view in profile, and SEM images of **C** head in profile **D** close-up of mandibles **E** close-up of spatulate setae.

**Taxonomic history.**Kempf (1972), Bolton (1995, 2014), Bolton et al. (2006).

**Distribution.** Afrotropical, Australasia, Indomalaya, Malagasy, Nearctic, Neotropical, Oceania, Palearctic.

**Galápagos distribution.** Introduced: Floreana, Isabela (SN, VA), San Cristóbal, Santa Cruz (Herrera et al. 2020).

***Strumigenyslouisianae* Roger, 1863**

Fig. 35

**Remarks.** Cited in Lubin (1984), (Pezzatti et al. (1998), von Aesch (2006) [ICCDRS], Causton et al. (2006), Herrera et al. (2014), Dekoninck et al. (2014) [ICCDRS, RBINS], Wauters et al. (2016), Herrera et al. (2020), Herrera (2015, 2019) and Herrera et al. (2020) [ICCDRS].

**Figure 35.**
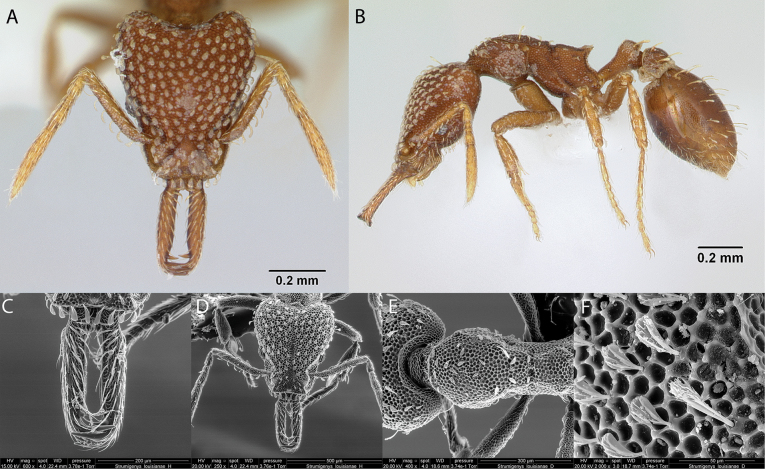
*Strumigenyslouisianae* worker micrographs in **A** head in full-face view **B** view in profile, and SEM images of **C** close-up on mandibles **D** head in full-face view **E** mesosoma in dorsal view **F** close-up on spatulate setae.

**Taxonomic history.** In Kempf (1972), Brandão (1991), Bolton (1995, 2014), Bolton et al. (2006).

**Distribution.** Nearctic, Neotropical.

**Galápagos distribution.** Introduced: Floreana, Isabela (CA, SN, VA), San Cristóbal, Santa Cruz (Herrera et al. 2020).

**New record.** Santiago.

***Strumigenysmembranifera* Emery, 1869**

Fig. 36

**Remarks.** Galápagos first published record in Herrera et al. (2014). Cited in Herrera (2015, 2019) and Herrera et al. (2020) [ICCDRS].

**Figure 36.**
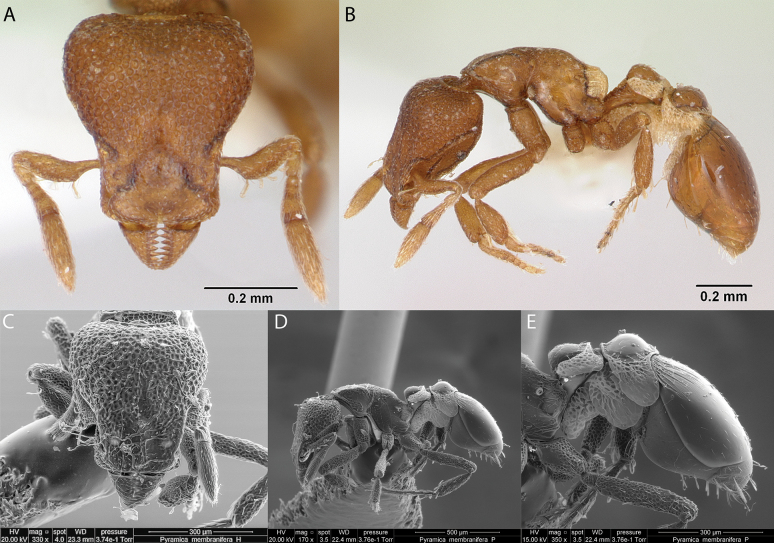
*Strumigenysmembranifera* worker micrographs in **A** head in full-face view **B** view in profile, and SEM images of **C** head in full-face view **D** head in profile **E** petiole and postpetiole with developed spongiform tissue.

**Taxonomic history.** In Kempf (1972), Bolton (1995, 2014), Bolton et al. (2006).

**Distribution.** Afrotropical, Australasia, Indomalaya, Malagasy, Nearctic, Neotropical, Oceania, Palearctic.

**Galápagos distribution.** Introduced: Isabela (VA, VW), Santiago (Herrera et al. 2020).

### ﻿Genus *Tetramorium* Mayr, 1855

***Tetramoriumbicarinatum* (Nylander, 1846)**

Fig. 37

**Remarks.** Originally cited as *Myrmicabicarinatum* in (Nylander, 1846). Cited as *Tetramoriumguineense* in Emery (1893), Wheeler (1919) [CAS], Wheeler (1924), Wheeler (1933) [CAS], Linsley and Usinger (1966), Kempf (1972), Clark et al. (1982), Brandão and Paiva (1994). As *T.bicarinaum* in Lubin (1984), Lubin (1985) [QCAZ], Abedrabbo (1994) [ICCDRS], de la Vega (1994), Meier (1994) [ICCDRS], Pezzatti et al. (1998) [ICCDRS], von Aesch and Cherix (2005), von Aesch (2006) [ICCDRS], Causton et al. (2006), Herrera and Causton (2010) [ICCDRS], Dekoninck et al. (2014) [ICCDRS, RBINS], Wauters et al. (2016) [RBINS], Herrera (2015, 2019), and Herrera et al. (2020) [ICCDRS].

**Figure 37.**
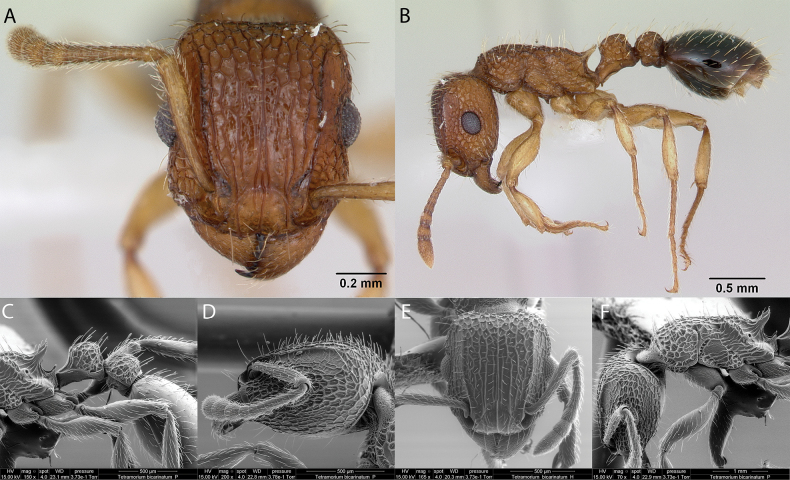
*Tetramoriumbicarinatum* worker micrographs in **A** head in full-face view **B** view in profile, and SEM images of **C** petiole and postpetiole in profile **D** head in profile **E** head in full-face view **F** mesosoma in profile.

**Taxonomic history.**Kempf (1972), Brandão (1991), Bolton (1995, 2014), Bolton et al. (2006).

**Distribution.** Afrotropical, Australasia, Indomalaya, Malagasy, Nearctic, Neotropical, Oceania, Palearctic.

**Galápagos distribution.** Introduced: Bainbridge #1, Bainbridge #2, Bainbridge #3, Bainbridge #4, Bainbridge #5, Bainbridge #6, Bainbridge #8, Baltra, Bar, Bayas, Caldwell, Daphne Mayor, Española, Fernandina, Floreana, Gardner (next to Floreana), Gardner (next to Española), Genovesa, Guy Fawkes, Isabela (CA, SN, VA, VD, VE, VW), Mariela Grande, Mariela Mediana, Marchena, Pinzón, Plaza Norte, Plaza Sur, Rábida, San Cristóbal, Santa Cruz, Seymour Norte, Santa Fé, Sombrero Chino (Herrera et al. 2020).

**New records.** Beagle #2, Beagle #3, Santiago Island.

***Tetramoriumcaldarium* (Roger, 1857)**

Fig. 38

**Remarks.** Originally cited as *Tetrogmuscaldarium* in (Roger, 1857). Cited in Brandão and Paiva (1994), Meier (1994), Pezzatti et al. (1998), von Aesch and Cherix (2005), von Aesch (2006), Causton et al. (2006), Dekoninck et al. (2014) [RBINS], Wauters et al. (2016) [RBINS], Herrera (2015, 2019), and Herrera et al. (2020) [ICCDRS].

**Figure 38.**
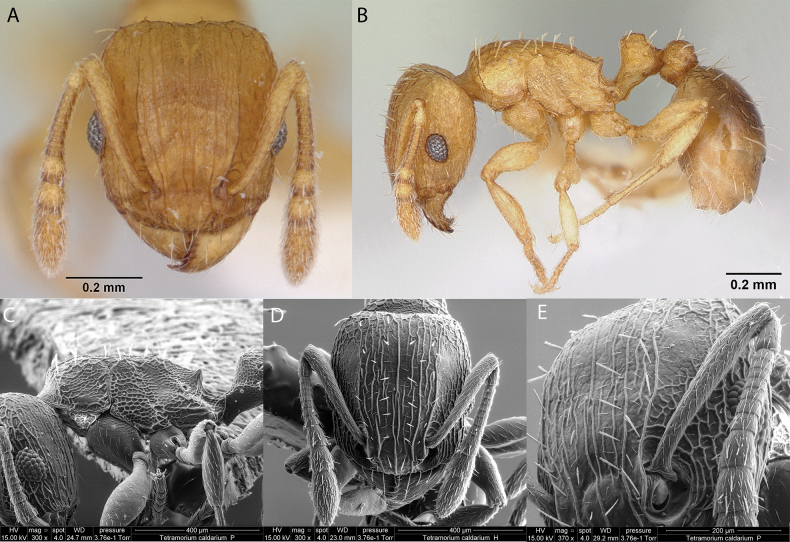
*Tetramoriumcaldarium* worker micrographs in **A** head in full-face view **B** view in profile, and SEM images of **C** mesosoma profile **D** head in full-face view **E** close-up on the antennal scrobe.

**Taxonomic history.**Kempf (1972), Brandão (1991), Bolton (1995, 2014), Bolton et al. (2006).

**Distribution.** Afrotropical, Australasia, Indomalaya, Malagasy, Nearctic, Neotropical, Oceania, Palearctic.

**Galápagos distribution.** Introduced: Floreana, Santa Cruz (Herrera et al. 2020).

***Tetramoriumlanuginosum* Mayr, 1870**

Fig. 39

**Remarks.** First published record (Pezzatti et al. (1998) [ICCDRS]. Cited also in Causton et al. (2006), Herrera and Causton (2010) [ICCDRS], Herrera (2015, 2019), and Herrera et al. (2020) [ICCDRS].

**Figure 39.**
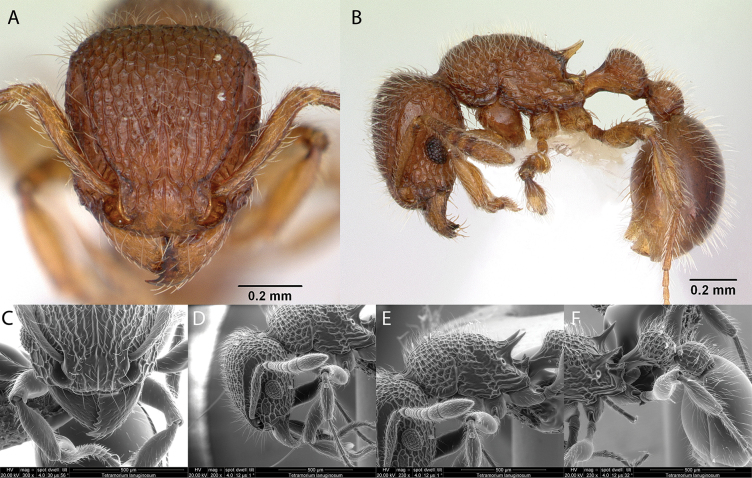
*Tetramoriumlanuginosum* worker micrographs in **A** head in full-face view **B** view in profile, and SEM images of **C** close-up on mandibles and clypeus **D** head in profile **E** mesosoma in profile **F** petiole and postpetiole in profile.

**Taxonomic history.** In Bolton (1995, 2014), Bolton et al. (2006).

**Distribution.** Afrotropical, Australasia, Indomalaya, Malagasy, Nearctic, Neotropical, Oceania, Palearctic.

**Galápagos distribution.** Introduced: Bainbridge #3, Bainbridge #8, Baltra, Floreana, Gardner (next to Española), Isabela (VD), Pinzón, Plaza Norte, Plaza Sur, Rábida, San Cristóbal, Santa Cruz, Santa Fé, Seymour Norte, Wolf (Herrera et al. 2020).

**New records.** Bainbridge #1, Bartolomé, Beagle #2, Beagle #3, Champion, Mao, Marchena, Santiago, Sombrero Chino.

***Tetramoriumsimillimum* (Smith, 1851)**

Fig. 40

**Remarks.** Originally cited as *Myrmicasimillimum* in (Smith, 1851). First published record in Wheeler (1919). Cited also in Wheeler (1933), Kempf (1972), Linsley and Usinger (1966), Clark et al. (1982), Lubin (1984), Lubin (1985), Brandão and Paiva (1994), Abedrabbo (1994) [ICCDRS], Peck et al. (1998), (Pezzatti et al. (1998) [ICCDRS], Roque-Albelo et al. (2000) [ICCDRS], von Aesch and Cherix (2005), von Aesch (2006) [ICCDRS], Causton et al. (2006), Herrera and Causton (2010) [ICCDRS], Herrera et al. (2014). Wauters et al. (2016) [RBINS], Herrera (2015, 2019), and Herrera et al. (2020) [ICCDRS].

**Figure 40.**
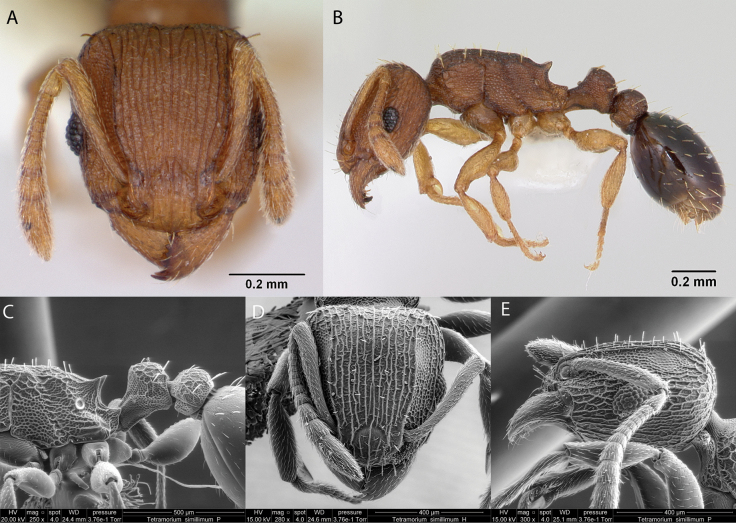
*Tetramoriumsimillimum* worker micrographs in **A** head in full-face view **B** view in profile, and SEM images of **C** petiole and postpetiole in profile **D** head in full-face view **E** head in profile.

**Taxonomic history.**Bolton (1995, 2014), Bolton et al. (2006).

**Distribution.** Afrotropical, Australasia, Indomalaya, Malagasy, Nearctic, Neotropical, Oceania, Palearctic.

**Galápagos distribution.** Introduced: Bainbridge #6, Baltra, Bar, Cousin, Daphne Mayor, Floreana, Gardner (next to Floreana), Isabela (SN, VA), Marchena, Mariela Grande, Santiago, San Cristóbal, Santa Cruz, Tortuga (Herrera et al. 2020).

**New record.** Mariela Mediana.

***Tetramoriumlucayanum* Wheeler, 1905**

Fig. 41

**Remarks.** First published record in Herrera et al. (2014), Cited also in Herrera (2015, 2019) and Herrera et al. (2020) [ICCDRS].

**Figure 41.**
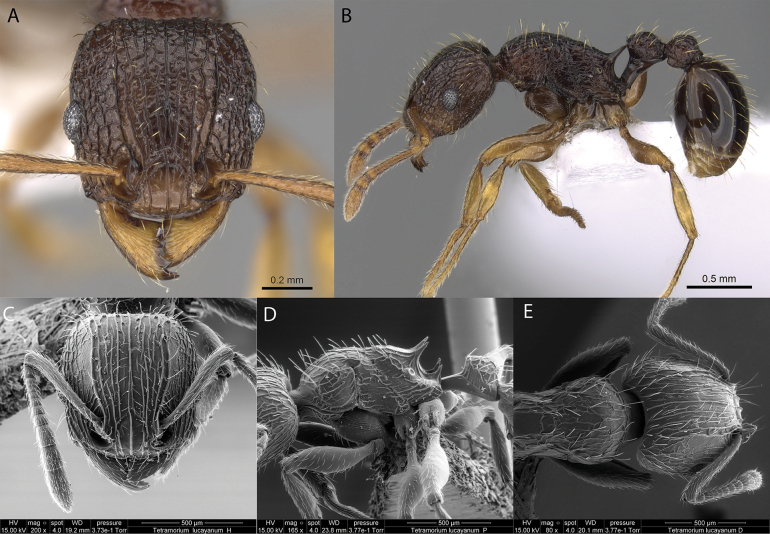
*Tetramoriumlucayanum* worker micrographs in **A** head in full-face view **B** view in profile, and SEM images of **C** head in full-face view **D** mesosoma in profile **E** head in dorsal view.

**Taxonomic history.**Bolton (1995, 2014), Bolton et al. (2006).

**Distribution.** Afrotropical, Neotropical, Palearctic.

**Galápagos distribution.** Introduced: Isabela (CA) (Herrera et al. 2014).

**New record.** Isabela (SN).

### ﻿Genus *Trichomyrmex* Mayr, 1855

***Trichomyrmexdestructor* (Jerdon, 1851)**

Fig. 42

**Remarks.** Originally cited as *Attadestructor* in (Jerdon, 1851). Cited as *Monomoriumdestructor*, in Pezzatti et al. (1998), von Aesch and Cherix (2005), von Aesch (2006), Causton et al. (2006), Herrera and Causton (2010) [ICCDRS], Herrera (2015, 2019), and Herrera et al. (2020) [ICCDRS].

**Figure 42.**
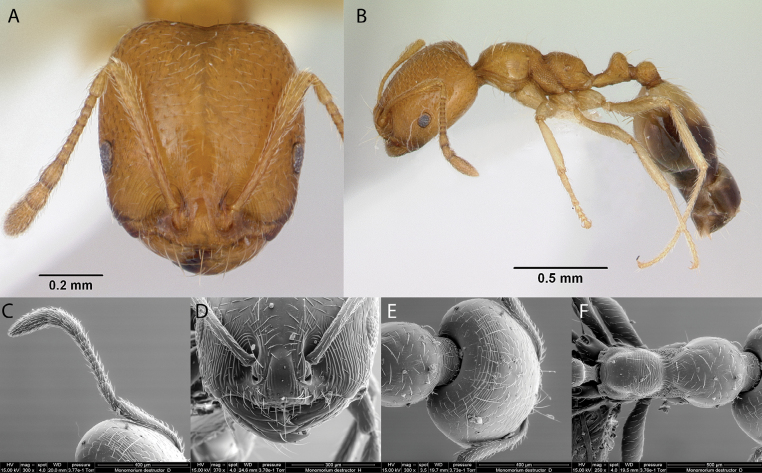
*Trichomyrmexdestructor* worker micrographs in **A** head in full-face view **B** view in profile, and SEM images of **C** close-up on antennae **D** close-up on mandibles **E** head in dorsal view **F** mesosoma in dorsal view.

**Taxonomic history.**Bolton (1995, 2014), Bolton et al. (2006).

**Distribution.** Afrotropical, Australasia, Indomalaya, Malagasy, Nearctic, Neotropical, Oceania, Palearctic.

**Galápagos distribution.** Introduced: Baltra, Floreana, Isabela (SN), Santiago (Herrera et al. 2020).

### ﻿Genus *Wasmannia* Forel, 1893

***Wasmanniaauropunctata* (Roger, 1863)**

Fig. 43

**Remarks.** Originally cited as *Tetramoriumauropunctata* in (Roger, 1863). First published record in Silberglied (1972). Cited in Lubin (1983), Lubin (1984) [ICCDRS], Lubin (1985), McMullen (1987), Williams (1987), Ulloa-Chacón et al. (1991), Coppois and Wells (1987), McMullen (1990), Brandão (1991), Williams and Whelan (1992), McMullen (1993), Brandão and Paiva (1994), Meier (1994), Abedrabbo (1994) [ICCDRS], Ulloa-Chacón and Cherix (1994), de la Vega (1994), Lundh (1998), Peck et al. (1998), Pezzatti et al. (1998) [ICCDRS], Roque-Albelo et al. (2000) [ICCDRS], Boada (2005) [ICCDRS], Causton et al. (2005) [ICCDRS], von Aesch (2006) [ICCDRS], Causton et al. (2006), McMullen (2007), Herrera and Causton (2010) [ICCDRS], Herrera and Longino (2008), (McMullen (2011), Herrera et al. (2013), Dekoninck et al. (2014) [ICCDRS, RBINS], Wauters et al. (2014) [RBINS], Wauters et al. (2016) [RBINS], Herrera (2015, 2019), and Herrera et al. (2020) [ICCDRS].

**Figure 43.**
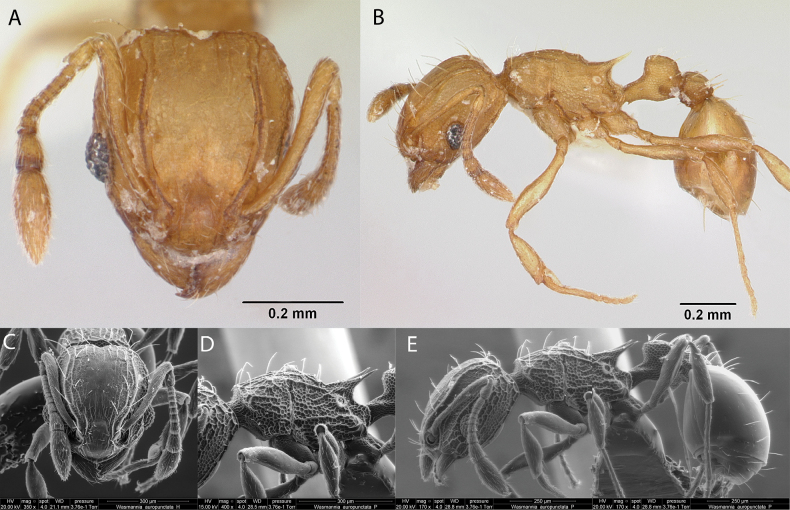
*Wasmanniaauropunctata* worker micrographs in **A** head in full-face view **B** view in profile, and SEM images of **C** head in full-face view **D** mesosoma in profile **E** head in profile **F** metasoma in profile.

**Taxonomic history.**Bolton (1995, 2014), Bolton et al. (2006).

**Distribution.** Afrotropical, Australasia, Indomalaya, Nearctic, Neotropical, Oceania, Palearctic.

**Galápagos distribution.** Introduced: Albany, Bainbridge #1, Baltra, Bowditch South, Champion, Cousin, Eden, Española, Floreana, Gran Felipe, Isabela (SN, VA, VD, VE, VW), Mao, Marchena, Pinzón, Rábida, Santiago, San Cristóbal, Santa Cruz, Seymour Norte, Santa Fé, Tortuga (Herrera et al. 2020).

### ﻿Key to the genera and species of the subfamily Ponerinae

**Table:** 

1	Mandible elongate and linear (Figs 49C, 50C); petiolar node armed with apical spine (Figs 49D, 50D). (*Odontomachus*)	**2**
–	Mandibles not elongate (Figs 44D, 47C, 48C); petiole not armed with apical spine (Figs 44C, B, 47A, C)	**3**
2	Entirely dark brown (Fig. 49A, B); long hairs located below the mandibles, running from the base towards the apex (Fig. 49C); anterior face of the petiole somewhat convex (Fig. 49D)	***Odontomachusbauri***
–	Somewhat tricolored: head, antennae and legs orangish, mesosoma reddish brown and gaster dark brown (Fig. 50A, B); ventral face of mandibles with short hairs running from base towards apex (Fig. 50C); anterior face of petiole almost straight or less convex than above (Fig. 50D)	***Odontomachusruginodis***
3	Mandibles falcate with apical tooth; anterior margin of clypeus triangular with carina conspicuously or slightly visible in median portion (Figs 47C, 48C); anterior legs with finely pectinate tarsal claws (Fig. 48D) (*Leptogenys*)	**4**
–	Triangular with dentate mandibles; anterior margin of clypeus without median carina (Fig. 44D; 46D); legs with simple tarsal claws (Fig. 44F) (*Hypoponera*)	**5**
4	Mandibles with basal margin distant from the anterior margin of clypeus when closed (Fig. 47A, C); in lateral view, mesosoma with numerous hairs and petiole higher than wide (Fig. 47D); ~ 5 mm long; body entirely brown (Fig. 47A, B)	***Leptogenyssantacruzi***
–	Mandibles with basal margin almost flush with the anterior border of clypeus when closed (Fig. 48A, C); mesosoma smooth and shiny with few setae; lacking longitudinal striae in the pronotum and propodeum; petiole elongated in lateral view (Fig. 48B, E); ~ 4 mm long; body black, mandibles, legs, and antennae brown (Fig. 48A, B)	**Leptogenyscf.gorgona**
5	Color dark red brown to black (Fig. 45A, B); scape of antenna reaching the occipital margin of the head (Fig. 45A); petiolar node quadrate (Fig. 45B, C)	***Hypoponeraopaciceps***
–	Color red brown to dark brown (Figs 44A, B, 46A, B); scape of antenna never reaching the occipital margin of the head (Figs 44A, 46E); petiolar node never quadrate	**6**
6	Lateral surface of petiole relatively coarse with the dorsum somewhat rounded, not totally covered with fine appressed hairs (Fig. 44C)	***Hypoponerabeebei***
–	Lateral surface of petiole somewhat more thick than coarse, with the dorsum somewhat triangular, sometimes covered by many fine appressed hairs (Fig. 46F)	***Hypoponeraopacior***

### ﻿Genus *Hypoponera* Santschi, 1938

***Hypoponerabeebei* (Wheeler, 1924)**

Fig. 44

**Remarks.** Originally cited as *Ponerabeebei* in Wheeler (1924: 107). *Hypoponerabeebei* in Linsley and Usinger (1966), Kempf (1972), Lubin (1985), Peck (1994a), Peck (1994b), Roque-Albelo et al. (2000), Wauters et al. (2016), Lubin (1984), Herrera (2015, 2019) and Herrera et al. (2020) [ICCDRS].

**Figure 44.**
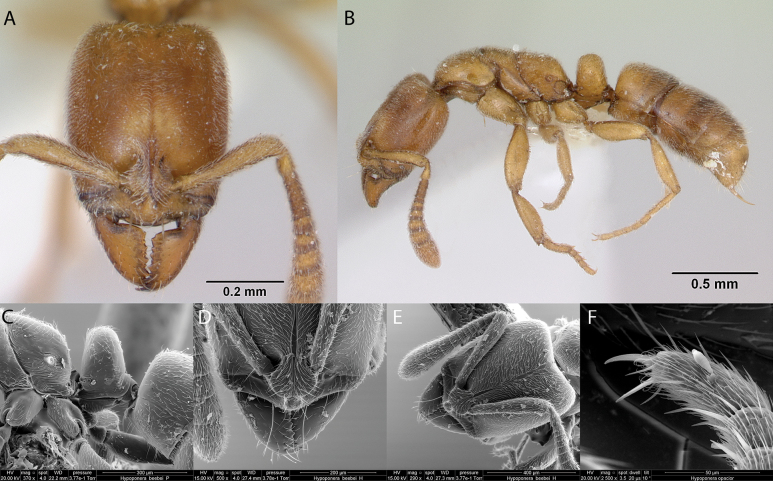
*Hypoponerabeebei* worker micrographs in **A** head in full-face view **B** view in profile, and SEM images of **C** close-up on petiole in profile **D** close-up on mandibles **E** head in full-face view **F** close-up on tarsal claws.

**Taxonomic history.**Kempf (1972), Bolton (1995, 2014), Bolton et al. (2006).

**Distribution.** Possibly endemic: Fernandina, Floreana, Isabela (CA, SN, VA, VW), Marchena, San Cristóbal, Santa Cruz, Seymour Norte, Genovesa (Herrera et al. 2020).

***Hypoponeraopaciceps* (Mayr, 1887)**

Fig. 45

**Remarks.** Originally cited as *Poneraopaciceps* in (Mayr, 1887). First published record in (Lubin 1983). Cited also in Lubin (1984), Peck (1994b), Dekoninck et al. (2014), Herrera (2015), Wauters et al. (2016), and Herrera et al. (2020).

**Figure 45.**
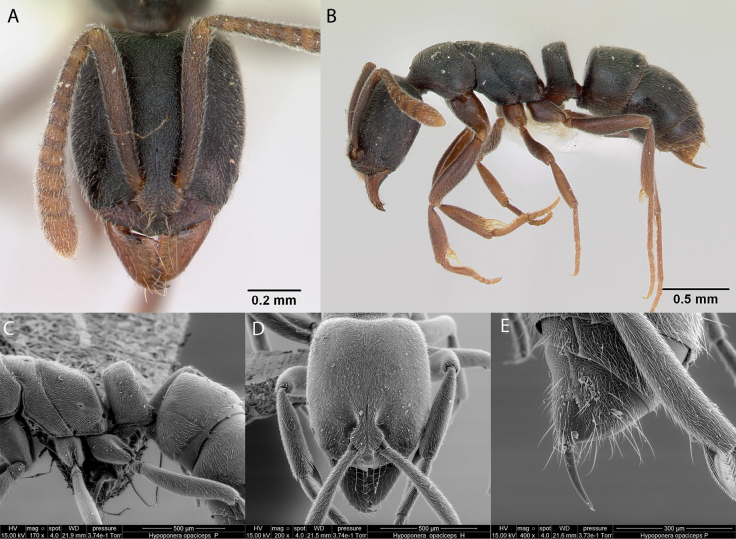
*Hypoponeraopaciceps* worker micrographs in **A** head in full-face view **B** view in profile, and SEM images of **C** close-up on petiole in profile **D** head in full-face view **E** stinging apparatus.

**Taxonomic history.**Bolton (1995, 2014), Bolton et al. (2006).

**Distribution.** Australasia, Indomalaya, Nearctic, Neotropical, Oceania, Palearctic.

**Galápagos distribution.** Introduced: Baltra, Fernandina, Floreana, Isabela (CA, SN, VA, VD) Marchena, San Cristóbal, Santa Cruz, Santiago (Herrera et al. 2020).

**Hypoponeracf.opacior (Forel, 1893)**

Fig. 46

**Remarks.** Originally cited as *Poneraopacior* (Forel, 1893). In Herrera et al. (2014), Dekoninck et al. (2014) [RBINS], Herrera (2015, 2019), and Herrera et al. (2020) [ICCDRS].

**Figure 46.**
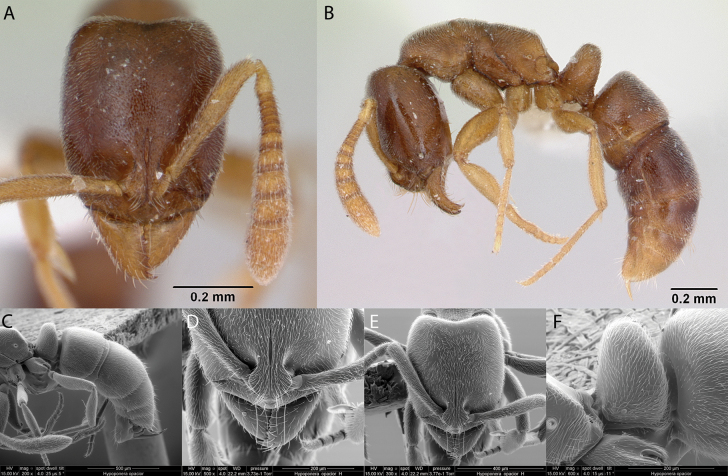
Hypoponeracf.opacior worker micrographs in **A** head in full-face view **B** view in profile, and SEM images of **C** metasoma in profile **D** close-up on mandibles **E** head in full-face view **F** close-up on petiole in profile.

**Taxonomic history.**Bolton (1995, 2014), Bolton et al. (2006).

**Distribution.** Nearctic, Neotropical.

**Galápagos distribution.** Introduced: Fernandina, Floreana, Isabela (CA, SN, VA, VD, VW), San Cristóbal, Santa Cruz (Herrera et al. 2014).

### ﻿Genus *Leptogenys* Roger, 1861

***Leptogenyssantacruzi* Lattke, 2011**

Fig. 47

**Remarks.** Cited in Herrera (2015, 2019) and Herrera et al. (2020) [CAS. ICCDRS].

**Figure 47.**
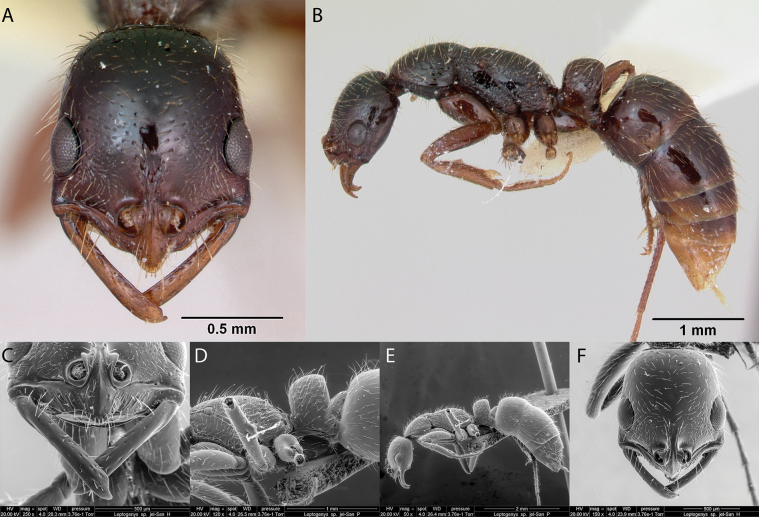
*Leptogenyssantacruzi* worker micrographs in **A** head in full-face view **B** view in profile, and SEM images of **C** close-up on mandibles **D** close-up on petiole in profile **E** view in profile **F** head in full-face view.

**Taxonomic history.**Lattke (2011) and Bolton (2014).

**Distribution.** Endemic: Isabela (VA), Santa Cruz Islands (Herrera et al. 2020).

**Leptogenys sp. gorgona (hh03)**

Fig. 48

**Remarks.** Cited in Lattke (2011), Herrera (2015, 2019) and Herrera et al. (2020) [ICCDRS].

**Figure 48.**
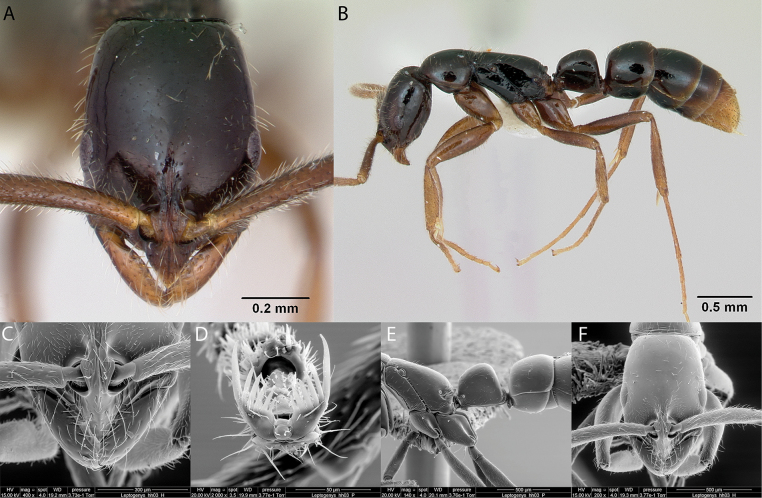
Leptogenyscf.gorgona worker micrographs in **A** head in full-face view **B** view in profile, and SEM images of **C** close-up on clypeus **D** close-up on tarsal claws **E** close-up on petiole in profile **F** head in full-face view.

**Distribution.** Native: Santa Cruz.

**New record.** Isabela Island (SN).

### ﻿Genus *Odontomachus* Latreille, 1804

***Odontomachusbauri* Emery, 1892**

Fig. 49

**Remarks.** Cited as *O.bauri* in Emery (1893), as *Odontomachushaematodabauri* in Wheeler (1919) [CAS], Wheeler (1924), Wheeler (1933) [CAS], Kempf (1972) and *Odontomachushaematoda* in Stitz (1932). *Odontomachusbauri* in Pezzatti et al. (1998), von Aesch and Cherix (2005), Linsley and Usinger (1966), Lubin (1984), Brandão (1991), Brandão and Paiva (1994), de la Vega (1994), von Aesch and Cherix (2005), von Aesch (2006) [ICCDRS], Causton et al. (2006), Dekoninck et al. (2014) [RBINS], Wauters et al. (2014), Herrera (2015, 2019) and Herrera et al. (2020) [ICCDRS, ICCDRS].

**Figure 49.**
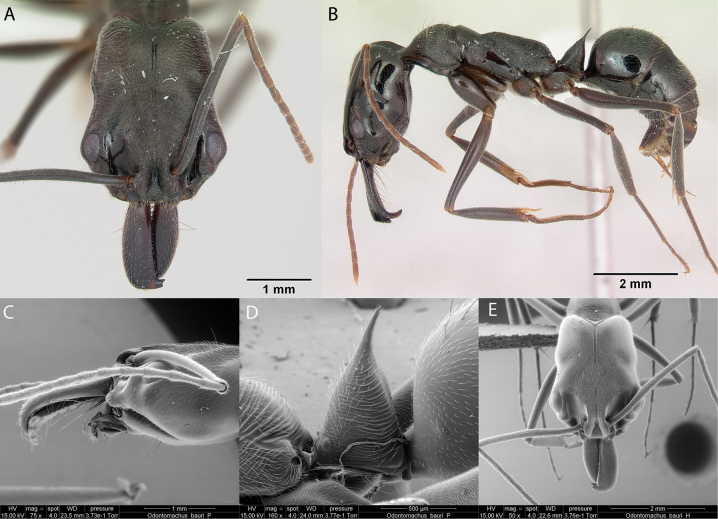
*Odontomachusbauri* worker micrographs in **A** head in full-face view **B** view in profile, and SEM images of **C** head in profile **D** close-up on petiole in profile **E** head in full-face view.

**Taxonomic history.**Bolton (1995, 2014), Bolton et al. (2006).

**Distribution.** Neotropical.

**Galápagos distribution.** Introduced: Floreana, Isabela (CA, SN), San Cristóbal, Santa Cruz (Herrera et al. 2020).

***Odontomachusruginodis* Wheeler, 1908**

Fig. 50

**Remarks.** First published record in Herrera et al. (2014), see also Herrera (2015, 2019) and (Herrera et al. 2020) [ICCDRS].

**Figure 50.**
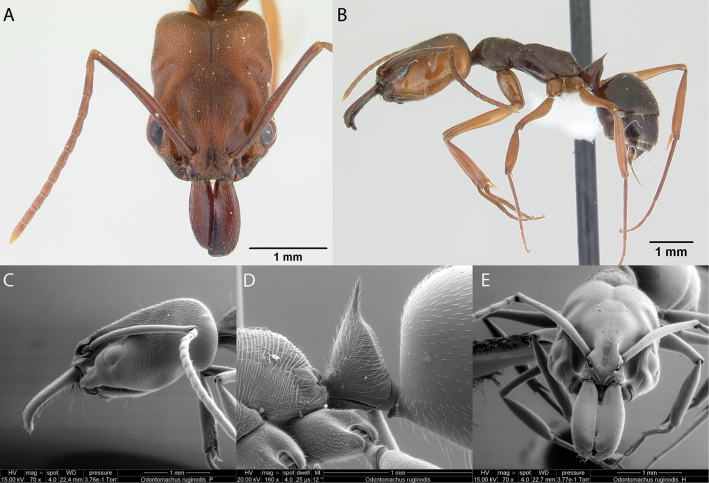
*Odontomachusruginodis* worker micrographs in **A** head in full-face view **B** view in profile, and SEM images of **C** head in profile **D** close-up on petiole in profile **E** head in full-face view.

**Taxonomic history.**Bolton (1995, 2014), Bolton et al. (2006).

**Distribution.** Nearctic, Neotropical.

**Galápagos distribution.** Introduced: Santa Cruz (Herrera et al. 2014).

## ﻿Additional information

### Author ORCIDs

Henri W. Herrera https://orcid.org/0000-0002-1417-4349

María C. Tocora https://orcid.org/0000-0002-5304-4010

Gianpiero Fiorentino https://orcid.org/0000-0001-6948-5032

Charlotte E. Causton https://orcid.org/0000-0002-2861-0025

Wouter Dekoninck https://orcid.org/0000-0002-7065-7310

Frederik Hendrickx https://orcid.org/0000-0002-1176-0318

### Data availability

All of the data that support the findings of this study are available in the main text.
